# Supplementary material for: Organophotoredox-Catalyzed Stereoselective Synthesis of Bicyclo[3.2.0]heptanes via [2+2] Photocycloaddition
Source: Molecules. 2025 May 8;30(10):2090. doi: 10.3390/molecules30102090 (PMC12113679; doi:10.3390/molecules30102090)
Supplement: Supplementary file 1 [file molecules-30-02090-s001.zip › molecules-3609083-supplementary.pdf]

SUPPORTING INFORMATION OF

Organophotoredox-Catalyzed Stereoselective Synthesis of Bi-cyclo[3.2.0]heptanes via [2+2] Photocycloaddition

Tommaso Benettin, Simonetta Resta, Alessandra Forni, Laura Raimondi, Alessandra Puglisi and Sergio Rossi*

Index

S1. General description of reagents and equipment **2**

S2. Synthesis of starting materials **3**

Synthesis of compound 2 3

Synthesis of compound 3 4

General synthesis A for chiral oxazolidinones derivatives 4a-b 4

Synthesis of (S)-4-(tertbutyl) oxazolidin-2-one 4a 4

Synthesis of (S)-4- phenyloxazolidin-2-one 4b 5

General procedure B for the synthesis of chloroacetyl derivatives 5a-b 5

(S)-4-tertbutyl-3-(2-chloroacetyl)oxazolidin-2-one 5a 5

(S)-4-phenyl-3-(2-chloroacetyl)-oxazolidin-2-one 5b 6

General Procedure C for the synthesis of Ylide derivates 6a-6b 6

(S)-4-(tert-butyl)-3-(2-(triphenylphosphaneylidene)acetyl)oxazolidin-2-one 6a 6

(S)-4-phenyl-3-(2-(triphenylphosphaneylidene)acetyl)oxazolidin-2-one 6b 7

General procedure D for the synthesis of amido-enones 7-8 7

Compound 7 7

Compound 8 8

General procedure E for the synthesis of *cis-anti* bicycle adducts 8

Compound 9a and 9b 8

Compound 10a and 10b 9

S3. Single crystal X-ray studies **10**

S4. Cyclic voltammetry (CV) experiment **10**

S5. NMR spectra **11**

S6. DFT calculations **17**

S7. Bibliography **58**

S1. General description of reagents and equipment

NMR spectra: ^{1}H-NMR spectra were recorded with instruments at 300 MHz (Brucker AV300). Chemical shifts are reported in ppm (δ), with the solvent reference relative to tetramethyl silane (TMS). ^{13}C-NMR spectra were recorded on a 300 MHz spectrometer (Bruker AV300) operating at 75 MHz, with complete proton decoupling. Carbon chemical shifts are reported in ppm (δ) relative to TMS with the respective solvent resonance as the internal standard ($CDCl_3$: δ = 77.0 ppm).

Mass spectra: High resolution mass spectra (HRMS) were acquired using a Bruker solariX XR Fourier transform ion cyclotron resonance mass spectrometer (Bruker Daltonik GmbH, Bremen, Germany) equipped with a 7 T refrigerated actively shielded superconducting magnet. The samples were ionized in positive ion mode using a MALDI or ESI ionization sources. Mass spectra have been also acquired using Advion, Expression^{S} CMS spectra. The samples were ionized in positive ion mode using a APCI or ESI ionization sources.

High Performance Liquid Chromatography (HPLC): HPLC analyses were performed with HPLC Agilent 1100 or 1200 series, using a Daicel CHIRALCEL OJ-H column. The specific operative conditions for each product are reported from time to time.

Chromatographic purification: The purification of products was performed by column chromatography with flash technique (according to the Still method) using as stationary phase silica gel 230-400 mesh from MERCK.

Thin-Layer Chromatography (TLC): Reactions and chromatographic purifications were monitored by analytical TLC using silica gel 60 F_{254} pre-coated glass plates and visualized using UV light (365nm) or $KMnO_4$ solution.

Electrochemical equipment: Cyclic voltammetry (CV) experiments were carried out using a Metrohm Autolab potentiostat/galvanostat. Data elaboration was performed with the NOVA software. The system features a three-electrode configuration with glassy carbon as working electrode, platinum wire as counter electrode and Ag/AgCl as reference electrode.

Home-made GREEN (530 nm) LEDs Photoreactor

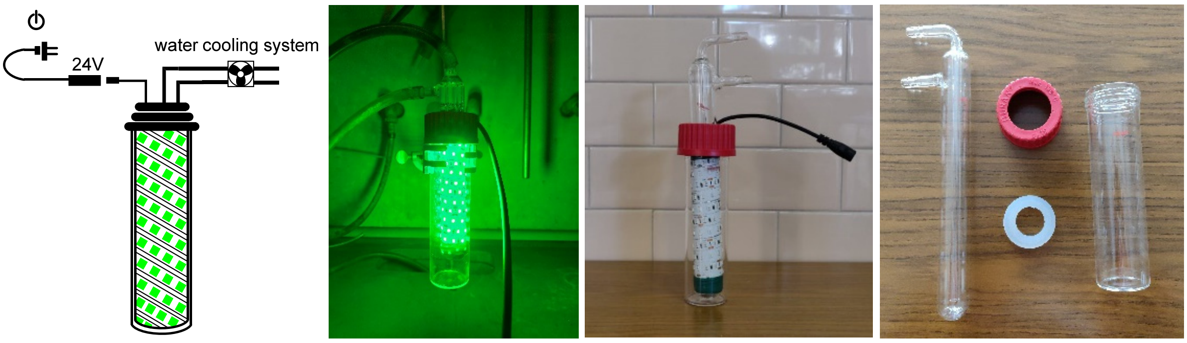

A B C D

Figure S1 - A) Water-cooled photoreactor scheme. B) Assembled photoreactor (on). C) Assembled photoreactor (off). D) Unassembled photoreactor (without LED strip).

<u>Construction of the Photoreactor</u>: A making-off video related to the construction of the photoreactor starting from a sublimator apparatus was already reported in literature.[1] The central sublimator glass-piece is first wrapped to the desired length with heavy duty aluminium foil to generate a socket for the LED-strip that possesses high heat conductive properties. Around this first layer is then coiled and glued (double sided adhesive tape) the LED-strip which is further secured in place at the top and bottom with electric isolating

tape. The cable is guided through the silicon rubber seal by puncturing it. The final reactor is then assembled as presented in Figure S1.

Green LEDs characterization: Commercially available SMD LED 2835 60 led/m 24V, with an IP95 "plug and play system" without extra wiring connection were employed in the realization of photoreactor showed in figure S1. The LEDs wavelength emission profile together with their specific light intensity (expressed as mW/cm^2) have been determined using a compact CCD spectrometer (model CCS200/M) connected to a multimode optical fibre, purchased by Thorlabs. As clearly depicted in Figure S2, green LEDs employed are characterized by an almost monochromatic emission profile showing a maximum of intensity located at ca. 512-514 nm. The light power intensity was thus checked using a Thorlabs PM200 power meter equipped with a S130VC power head with a Si detector. The measured light intensities, though slightly decreasing by moving the maximum of LEDs emission towards longer wavelengths, resulted to be I = 423.9 ± 0.6 mW/cm^2

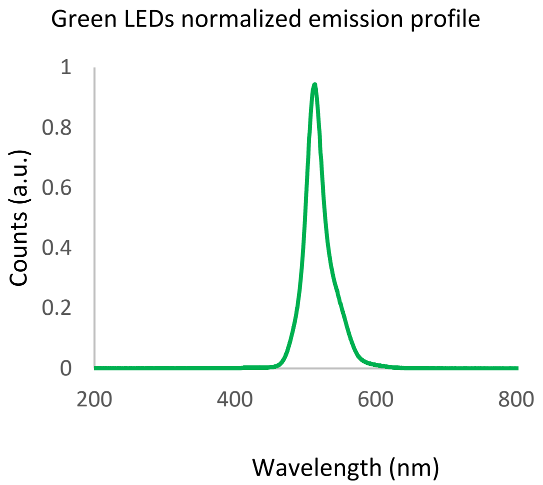

Figure S2 – Green LEDs Normalized emission profile.

S2. Synthesis of starting materials

Synthesis of compound 2

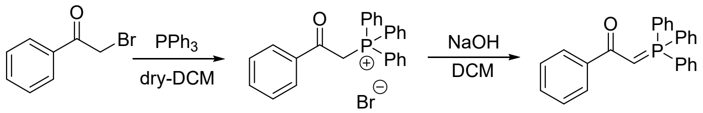

Step 1: In a round bottom flask, triphenylphosphine (1.343 g, 5.1 mmol, 1 equiv.) was dissolved in 5 mL of dry CH_2Cl_2 under N_2 atmosphere. A solution of α-bromoacetophenone (992 mg, 5 mmol, 1 equiv.) in 10 mL of dry CH_2Cl_2 was added and the reaction mixture was stirred for 24h at room temperature. After that time, the crude was evaporated under vacuo and washed with diethyl ether (3x15 mL) to remove any unreacted reagents or by-products. The phosphonium salt was isolated as a white powder and used in the further step without any purification. [NOTE: the phosphonium salt is stable and can be stored at rt].

Step 2: The phosphonium salt was dissolved in 10 mL of CH_2Cl_2 and 10 mL of a 2M NaOH solution was slowly added to the reaction mixture under vigorous stirring. The reaction was stirred vigorously for 3h, then the organic phase was separated, washed with brine (3x10 mL), dried over Na_2SO_4, filtered and concentrated under vacuo. The desired product 2 was obtained as a foaming sticky oil (1.7 g, 4.3 mmol, 86% yield). All analytical data are in agreement with literature.[1]

1H-NMR (300 MHz, Chloroform-d): δ 8.00 (d, J = 3.4 Hz, 2H), 7.82-7.65 (m, 4H), 7.65-7.41 (m, 6H), 7.41-7.32 (m, 2H), 7.28 (d, J = 1.4 Hz, 2H), 4.48 (s, 2H), 4.41 (s, 3H).

31P-NMR (122 MHz, Chloroform-d): δ 16.60.

Synthesis of compound 3

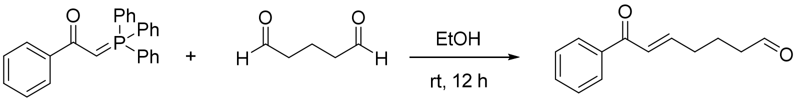

Compound **2** (1 equiv., 10.5 mmol, 4.0 g) was dissolved in EtOH (80 ml) at room temperature, then glutaric aldehyde (20 equiv. 0.2 mol 80 ml of a 2.6 M solution in water) was added. The reaction was stirred for 12h at rt. After this time, H_2O was added (200 mL) and the organic phase was extracted with Et_2O (3 X 200 ml). Organic layer was washed with HCl 0.2M (3 X 300 ml) and then with brine (3 X 50 ml). The organic layers were combined, dried over anhydrous sodium sulfate, filtered, and concentrated in vacuo. The crude compound was purified by silica gel chromatography (8/2 hexane/EtOAc) affording the pure product as a colourless oil in 75% yield (4,58 g). Analytical data are in agreement with those reported in literature.[2]

^{1}H NMR (300 MHz, Chloroform-d) δ: 9.81 (t, *J* = 1.4 Hz, 1H), 7.94 (dt, *J* = 8.4, 1.6 Hz, 2H), 7.59–7.55 (m, 1H), 7.51–7.46 (m, 2H), 7.03 (dt, *J* = 15.4, 6.8 Hz, 1H), 6.92 (dt, *J* = 15.4, 1.4 Hz, 1H), 2.54 (td, *J* = 7.2, 1.4 Hz, 2H), 2.42–2.35 (m, 2H), 1.89 (p, *J* = 7.3 Hz, 2H).

General synthesis A for chiral oxazolidinones derivatives 4a-b

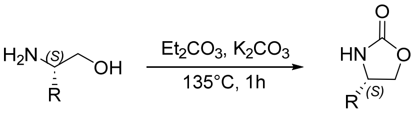

A 100 mL round-bottom flask, equipped with a Vigreux column and a magnetic stirring bar, was charged with the desired α-amino alcohol (1 equiv.), diethyl carbonate (2 equiv.), and potassium carbonate (1 equiv.). The reaction mixture was stirred and heated to 135 °C until ethanol was completely distilled off (approximately 1–2 hours). After completion, the mixture was cooled to room temperature, diluted with 40 mL of water, and extracted with CH_2Cl_2 (3 × 30 mL). Organic phaser was washed with brine (40 mL), dried over anhydrous sodium sulfate, filtered, and concentrated in vacuo.

Synthesis of (S)-4-(tertbutyl) oxazolidin-2-one 4a

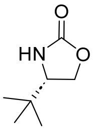

Compound 4a was synthetized according to the general procedure A, starting from (S)-2-amino-3,3-dimethylbutan-1-ol. The product was purified by flash column chromatography on silica gel to afford a white needle crystal in 53% yield (823 mg). Analytical data are in agreement with those reported in literature.[2]

Rf = 0.35 (eluent: hexane/ethyl acetate 1:1)

^{1}H NMR (400 MHz, Chloroform-d, ppm) δ 6.81 (br s, 1H), 4.36 (t, *J* = 8.8 Hz, 1H), 4.18 (dd, *J* = 8.8, 5.6 Hz, 1H), 3.60 (m, 1H), 0.91 (s, 9H).

Synthesis of (S)-4- phenyloxazolidin-2-one 4b

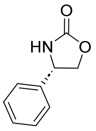

Compound 4b was synthetized according to the general procedure A, starting from (S)-2-amino-2-phenylethan-1-ol. The product was purified by flash column chromatography on silica gel to afford a white needle crystal in 78% yield (230 mg). Analytical data are in agreement with those reported in literature.[3]

Rf = 0.42 (eluent: hexane/ethyl acetate 1:1)

^{1}H NMR (Chloroform-d) ppm (δ): 2.84 (broad s., 3H, NH_2 + OH); 3.46 (m, $1H_a$, CH_2); 3.56 (m, $1H_b$, CH_2); 3.87 (m, 1H, CH); 7.20 (m, 5H, arom.).

General procedure B for the synthesis of chloroacetyl derivatives 5a-b

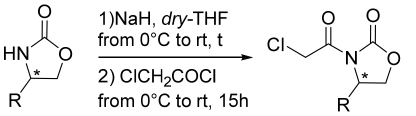

Freshly washed NaH (1.1 equiv., 2.11 mmol) was loaded in a heat gun dried Schlenk tube under nitrogen atmosphere, then freshly distilled THF was added (8 ml) and the mixture was cooled at 0 °C. After that, the desired oxazolidinone (1 equiv. 1.92 mmol) was added portion wise, and the reaction was warmed to room temperature. The mixture was stirred for 5 hours, then cooled at 0°C. Chloroacetyl chloride (1.7 equiv, 3,26 mmol) was then added dropwise, and the reaction mixture was stirred for 15 h at room temperature. After this time, the mixture was filtered on celite pad, and the solvent was evaporated under reduced pressure. The crude residue was then diluted in CH_2Cl_2, and the mixture was washed with 10% $NaHCO_3$ solution, dried over $MgSO_4$, filtered, and dried in vacuum. Target product was isolated after chromatographic purification using hexane:EtOAc 7:3 as eluent.

(S)-4-tertbutyl-3-(2-chloroacetyl)oxazolidin-2-one 5a

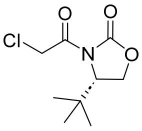

Synthetized according to the general procedure B, starting from (S)-4-(tertbutyl) oxazolidin-2-one. The product was purified by flash column chromatography on silica gel to afford a yellow oil in 76% yield (300 mg). Analytical data are in agreement with those reported in literature.[4]

Rf = 0.26 (eluent: hexane:ethyl acetate 8:2)

1H-NMR (300 MHz, Chloroform-d) = δ 4.73 (dd, J= 25 Hz, 15.46 Hz, 2H), 4.45 (dd, J= 6.96 Hz, 2.2Hz, 1H), 4.37 – 4.29 (m, 2H), 0.96 (s, 9H).

(S)-4-phenyl-3-(2-chloroacetyl)-oxazolidin-2-one 5b

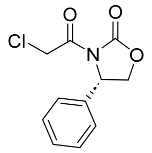

Synthetized according to the general procedure B, starting from (S)-4- phenyloxazolidin-2-one. The product was purified by flash column chromatography on silica gel to afford a white solid in 92% yield (1,243 g). Analytical data are in agreement with those reported in literature.[5]

Rf = 0.28 (eluent: hexane:ethyl acetate 7:3)

1H-NMR (300 MHz, Chloroform-d): δ 7.48-7.30 (m, 5H), 5.46 (dd, J = 8.7, 3.8 Hz, 1H), 4.84-4.69 (m, 3H), 4.39 (dt, J = 6.1, 3.0 Hz, 1H).

General Procedure C for the synthesis of Ylide derivates 6a-6b

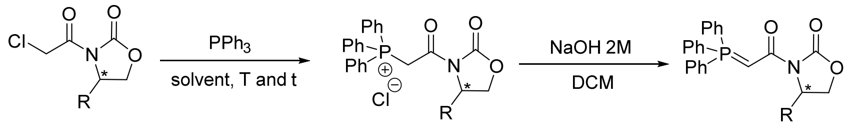

PPh_3 (1.1 equiv. 1.35 mmol) was added to a solution of the desired chloroacetyl derivate (1 equiv. 1.23 mmol) in degassed CH_2Cl_2 (10 ml) and the mixture was stirred at rt for 48h under nitrogen. The reaction mixture was concentrated in vacuo and the residue was dissolved in hot water (400 ml), 50°C. After the addition of 4M NaOH aqueous solution, the mixture was shacked for 5 minutes and immediately extracted with EtOAc and the organic layer washed with brine. The organic layers were combined, dried over anhydrous sodium sulfate, filtered, and concentrated in vacuo. Target product was isolated after chromatographic purification (typically 7/3 hexane/EtOAc).

(S)-4-(tert-butyl)-3-(2-(triphenylphosphaneylidene)acetyl)oxazolidin-2-one 6a

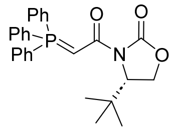

Synthetized according to the general procedure C starting from compound 5a. The product was purified by flash column chromatography on silica gel to afford a white oil in 75% yield (385 mg). Analytical data are in agreement with those reported in literature.[4]

1H-NMR (Chloroform-d) = δ 7.70-7.41 (m, 15H), 4.48 (dd, J= 7.33 Hz, 2.30 Hz, 1H), 4.22- 4.10 (m, 3H), 0.91 (s, 9H)

(S)-4-phenyl-3-(2-(triphenylphosphaneylidene)acetyl)oxazolidin-2-one 6b

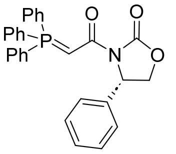

Synthetized according to the general procedure C starting from compound 5b. This compound wasn't purified and was directly used for the synthesis of compound **8**. Analytical data are in agreement with those reported in literature.[5]

1H-NMR (300 MHz, Chloroform-d): δ 7.53-7.28 (m, 20H), 5.59 (dd, J = 7.91, 2.53 Hz, 1H), 4.62 (t, J = 7.34 Hz, 1H), 4.15 (dd, J = 8.23, 3.47 Hz, 1H).

31P-NMR (122 MHz, Chloroform-d): δ 18.18

General procedure D for the synthesis of amido-enones 7-8

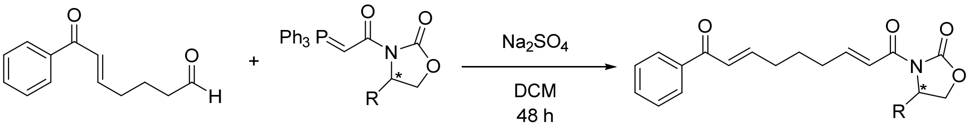

Aldehyde 3 (1 equiv., 0.35 mmol) and the desired ylide (1.5 equiv., 0.53 mmol) were dissolved in CH_2Cl_2 (0.5 M), then $MgSO_4$ (9 equiv, 3.15 mmol) was added to the stirred solution. The reaction was allowed to stir for 48h at rt. After this time, the mixture was poured in water, then extracted three times with CH_2Cl_2. The organic layers were combined, dried over anhydrous sodium sulfate, filtered, and concentrated in vacuo. Target product was purified by column chromatography using hexane:EtOAc 7:3 as eluent.

Compound 7

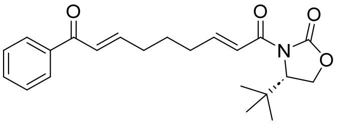

Prepared according to the general procedure C starting from 6a. The product was purified by flash column chromatography on silica gel to afford a transparent viscous oil in 70% yield (90mg). All analytical data are in agreement with literature.[6]

1H-NMR (300 MHz, Chloroform-d) = δ 7.90 (d, J = 7.4 Hz, 2H), 7.57 – 7.37 (m, 3H), 7.27 (d, J = 15.7 Hz, 1H), 7.16 – 6.95 (m, 2H), 6.88 (d, J = 15.6 Hz, 1H), 4.48 (dd, J = 7.2, 2.1 Hz, 1H), 4.31 – 4.15 (m, 2H), 2.41 – 2.26 (m, 5H), 1.71 (p, J = 7.4 Hz, 2H), 0.91 (s, 9H).

13C-NMR (75 MHz, Chloroform-d) = δ 190.8, 165.4, 154.7, 150.3, 148.6, 137.9, 132.9, 128.6, 126.6, 121.3, 65.3, 60.9, 36.0, 32.2, 32.1, 23.6, 25.7.

Compound 8

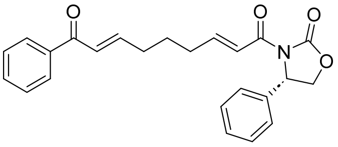

Prepared according to the general procedure C starting from 6b. The product was purified by flash column chromatography on silica gel to afford a transparent viscous oil in 50% yield (68 mg). All analytical data are in agreement with literature.[6]

1H NMR (300 MHz, Chloroform-d): δ 7.93 (t, J = 7.2 Hz, 1H), 7.62 – 7.24 (m, 8H), 7.18 – 6.84 (m, 4H), 5.50 (dt, J = 8.8, 4.4 Hz, 1H), 4.73 (t, J = 8.8 Hz, 1H), 4.30 (dt, J = 12.2, 6.1 Hz, 1H), 2.44 – 2.26 (m, 4H), 1.84 – 1.62 (m, 2H).

13C NMR (75 MHz, Chloroform-d) δ 199.2, 190.6, 150.6, 148.4, 139.0, 132.6, 129.1, 128.7, 128.5, 126.5, 125.9, 120.9, 69.9, 57.7, 32.1, 32.0, 29.7, 26.5.

General procedure E for the synthesis of *cis-anti* bicycle adducts

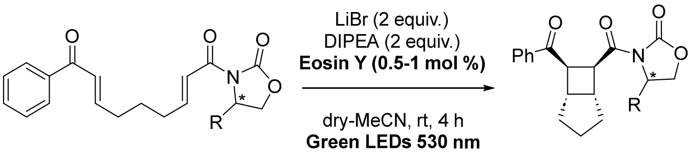

A dry 10 mL vial was charged with Eosin Y (1.35 µmol), LiBr (0.54 mmol, 2 equiv), dry acetonitrile (2.2 mL), and the desired aryl-enone (0.27 mmol, 1 equiv). The resulting solution was sonicated for 10 minutes, after which freshly distilled iPr_2NEt (0.54 mmol, 2 equiv) was added. The reaction mixture was then degassed by three freeze-pump-thaw cycles. The vial was placed at a 0 cm distance from the surface of the photoreactor and irradiated with green LEDs at room temperature under continuous stirring for 4 hours. After completion, the crude reaction mixture was directly loaded onto a silica gel column for purification.

Compound 9a and 9b

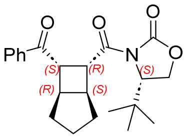

9a

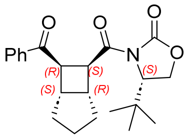

9b

Prepared according to the general procedure E starting from 7. The products were purified by flash column chromatography on silica gel (using hexane:EtOAc 9:1 as eluent) to afford compound **9a** as a white solid 38% yield (38 mg) and compound **9b** as transparent oil in 24% of yield (24 mg).

The overall yield is 62% with a d.r. of 62:38 (**9a:9b**)

9a:

Rf = 0.42 (Hex:EtOAc 90:10).

^{1}H-NMR (300 MHz, Chloroform-d): δ 7.81-7.78 (d, J=8.73 Hz, 2 H), 7.57-7.51 (t, J = 6.55, 7.28, 1 H), 7.46-7.40 (t, J=7.28 6.55 Hz, 2 H), 4.52-4.49 (dd, J=1.04, 7.55 Hz, 1 H), 4.30-4.17 (m, 2 H), 4.07-4.01 (dd, J=5.73, 9.38 Hz, 1 H), 3.62-3.56 (dd, J=5.73, 9.38 Hz, 1 H), 3.48-3.40 (q, J=7.31, 6.79, 7.31 Hz, 1 H), 2.05-1.88 (m, 3 H), 1.78-1.50 (m, 4 H), 0.93 (m, 9 H).

^{13}C-NMR (75 MHz, Chloroform-d): δ 199.2, 173.7, 155.9, 135.0, 133.0, 133.3, 128.6, 128.4, 65.5, 61.8, 49.6, 42.5, 40.3, 36.3, 32.4, 32.3, 25.7, 24.8.

HRMS calculated 392.1838; found 392.1847

<u>9b</u>:

Rf = 0.30 (Hex:EtOAc 90:10).

^{1}H-NMR (300 MHz, Chloroform-d): δ 7.81-7.78 (d, J=8.73 Hz, 2 H), 7.57-7.51 (t, J = 6.55, 7.28, 1 H), 7.46-7.40 (t, J=7.28 6.55 Hz, 2 H), 4.52-4.49 (dd, J=1.04, 7.55 Hz, 1 H), 4.30-4.17 (m, 2 H), 4.07-4.01 (dd, J=5.73, 9.38 Hz, 1 H), 3.62-3.56 (dd, J=5.73, 9.38 Hz, 1 H), 3.48-3.40 (q, J=7.31, 6.79, 7.31 Hz, 1 H), 2.05-1.88 (m, 3 H), 1.78-1.50 (m, 4 H), 0.93 (m, 9 H).

^{13}C-NMR (75 MHz, Chloroform-d): δ 199.4, 173.9, 154.4, 139.8, 133.2, 129.1, 128.7, 128.5, 128.2, 70.4, 58.0, 49.8, 42.6, 40.4, 35.8, 32.3, 32.2, 29.7, 24.8.

HRMS calculated 392.1838; found 392.1847

Compound 10a and 10b

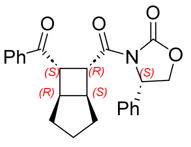

10a

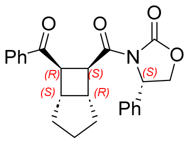

10b

Prepared according to the general procedure E starting from 8 using 1 mol% of photocatalyst. The products were purified by flash column chromatography on silica gel (using hexane:EtOAc 9:1 as eluent) to afford compound **10a** as a white solid 44% yield (44 mg) and compound **10b** as yellow oil in 13% of yield (13 mg).

The overall yield is 57% with a d.r. of 78:22 (**10a:10b**)

<u>10a</u>:

Rf = 0.36 (Hex:EtOAc 80:20).

^{1}H-NMR (300 MHz, Chloroform-d): δ 7.90-7.74 (d, J = 7.74, 2H), 7.59-7.52 (t, J = 7.81, 7.81 Hz, 1H), 7.47-7.27 (m, 7H), 5.59-5.52 (dd, J = 4.28, 8.82 Hz, 1H), 4.24-4.1 (m, 2H), 3.51-3.43 (m, 1H), 3.42-3.32(q, J = 7.64, 6.99, 6.03 Hz, 1H), 1.99-1.86(m, 2H), 1.66-1.42(m, 3H), 1.29-1.16(m, 2H).

^{13}C-NMR (75 MHz, Chloroform-d): δ 199.4, 173.9, 154.4, 139.8, 135.0, 133.2, 129.1, 128.7, 128.5, 128.4, 126.2, 70.4, 58.0, 49.8, 42.6, 40.4, 35.8, 32.3, 32.2, 29.7, 24.8.

HRMS calculated 389.1612; found 389.1644

<u>10b</u>:

Rf = 0.23 (Hex:EtOAc 80:20).

^{1}H-NMR (300 MHz, Chloroform-d): δ 8.05-7.98 (d, J=7.24, 2H), 7.98-7.93 (d, J=7.24, 2H), 7.59-7.43 (m, 6H), 4.59-4.52 (dd, J = 8.84, 10.36 Hz, 1H), 4.32-4.24 (t, J = 7.72, 1H), 3.31-3.19 (m, 1H), 3.10-3.02 (q, J = 6.63 Hz, 1H), 1.96-1.70 (m, 3H), 1.56-1.35 (m, 2H).

^{13}C-NMR (75 MHz, Chloroform-d): δ 199.8, 172.5, 153.9, 138.7, 135.2, 132.9, 128.8, 128.5, 128.5, 128.4, 126.6, 70.6, 60.4, 58.3, 49.6, 43.1, 40.1, 36.8, 32.2, 30.9, 29.7, 24.9, 21.1, 14.2.

HRMS calculated 389.1612; found 389.1644

S3. Single crystal X-ray studies

X-ray data of 9a have been collected on a Rigaku XtaLAB Synergy S X-ray diffractometer (Rigaku Co., Tokyo, Japan) operated with a mirror-monochromated microfocus Cu-Kα radiation (λ = 1.54184 Å) at 50 kV and 1.0 mA and equipped with a CCD HyPix 6000 detector. The structure has been solved using direct methods and refined with SHELXL-18 [14] using a full-matrix least squares procedure based on F2 using all data. Hydrogen atoms have been placed at geometrically estimated positions. Full details of crystal data and structure refinement, in CIF format, are available as Supplementary Information. CCDC reference number: 2442395.

S4. Cyclic voltammetry (CV) experiment

The CV of substrate **7** [5 mM] was recorded in 10 mL MeCN (0.2 M LiBr), using a glassy carbon as working electrode, Pt wire as counter and Ag/AgCl as reference electrodes, scan rate: 100 mV/s.

Two irreversible reduction peaks were found: P1 = 0.85 V and P2 = -1.13 V

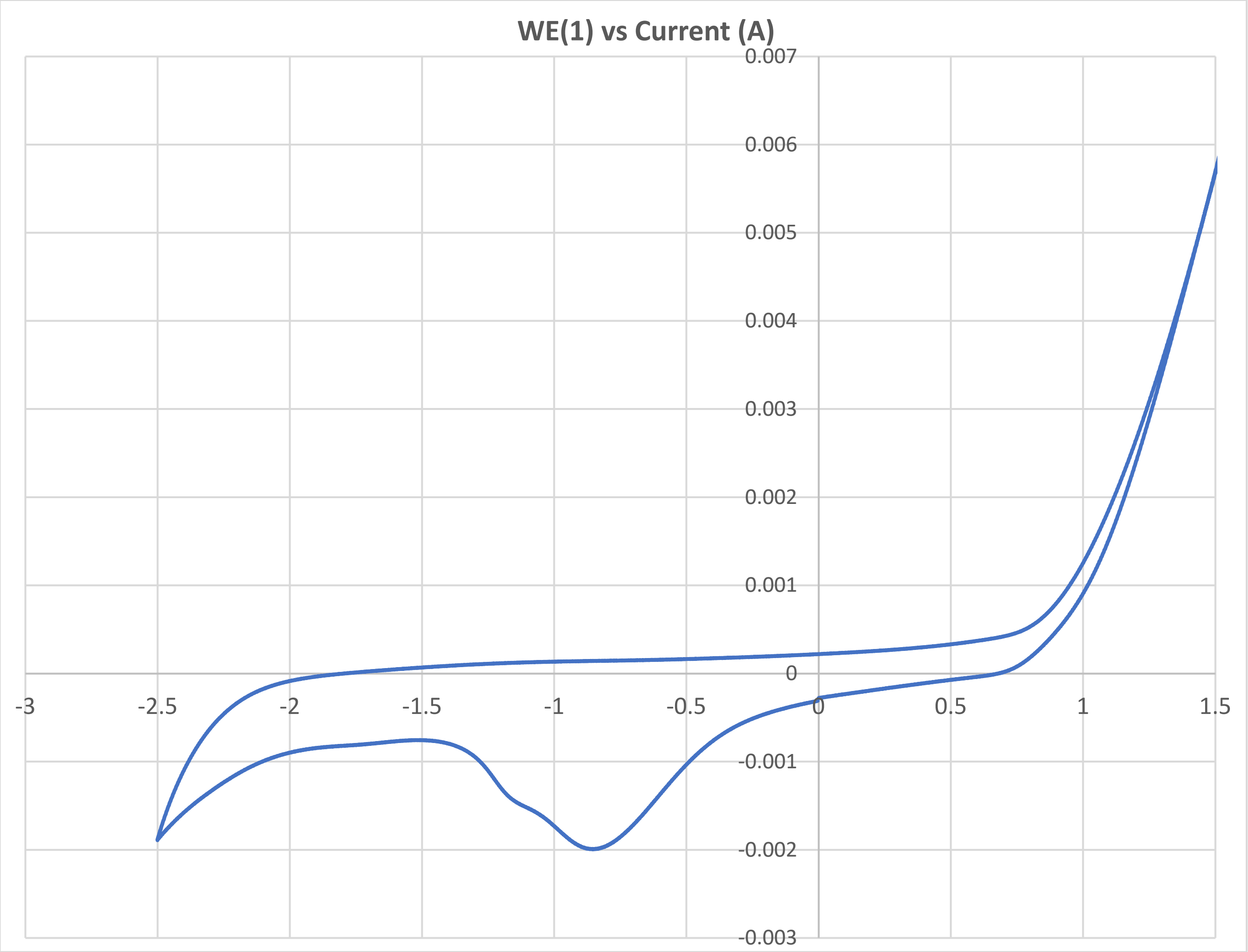

Figure S3. Cyclic voltammograms of substrate **7**

S5. NMR spectra

Compound 9a

1H-NMR:

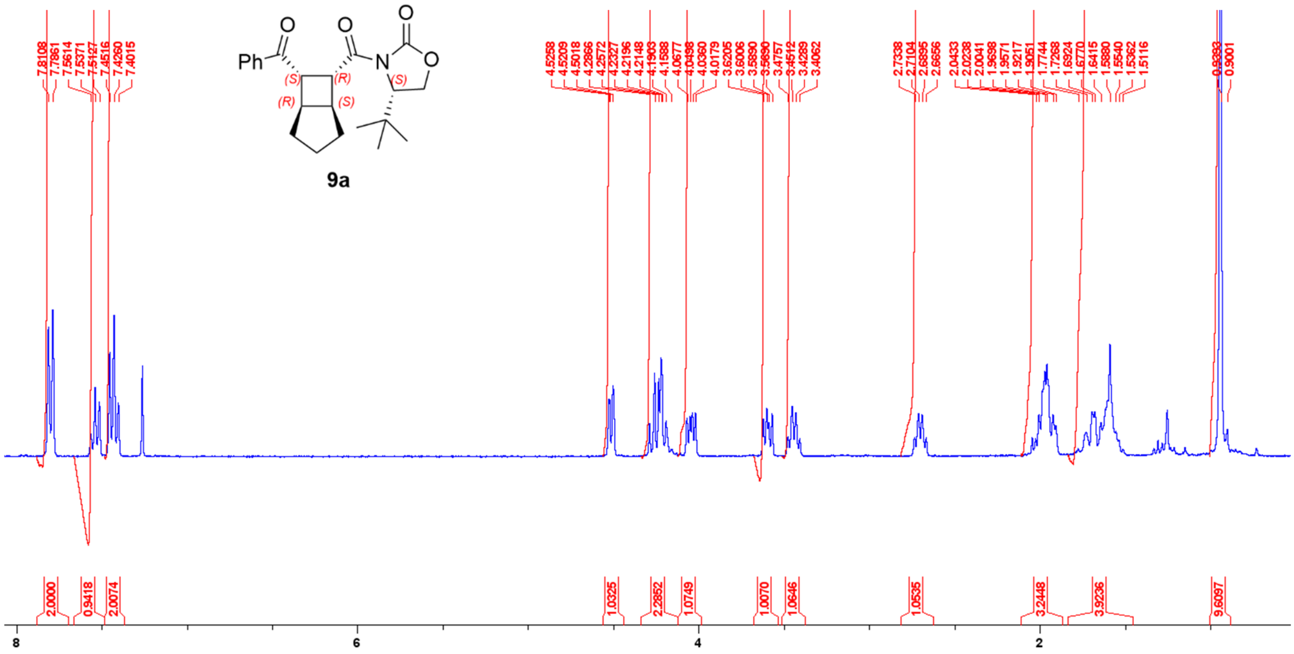

13C-NMR:

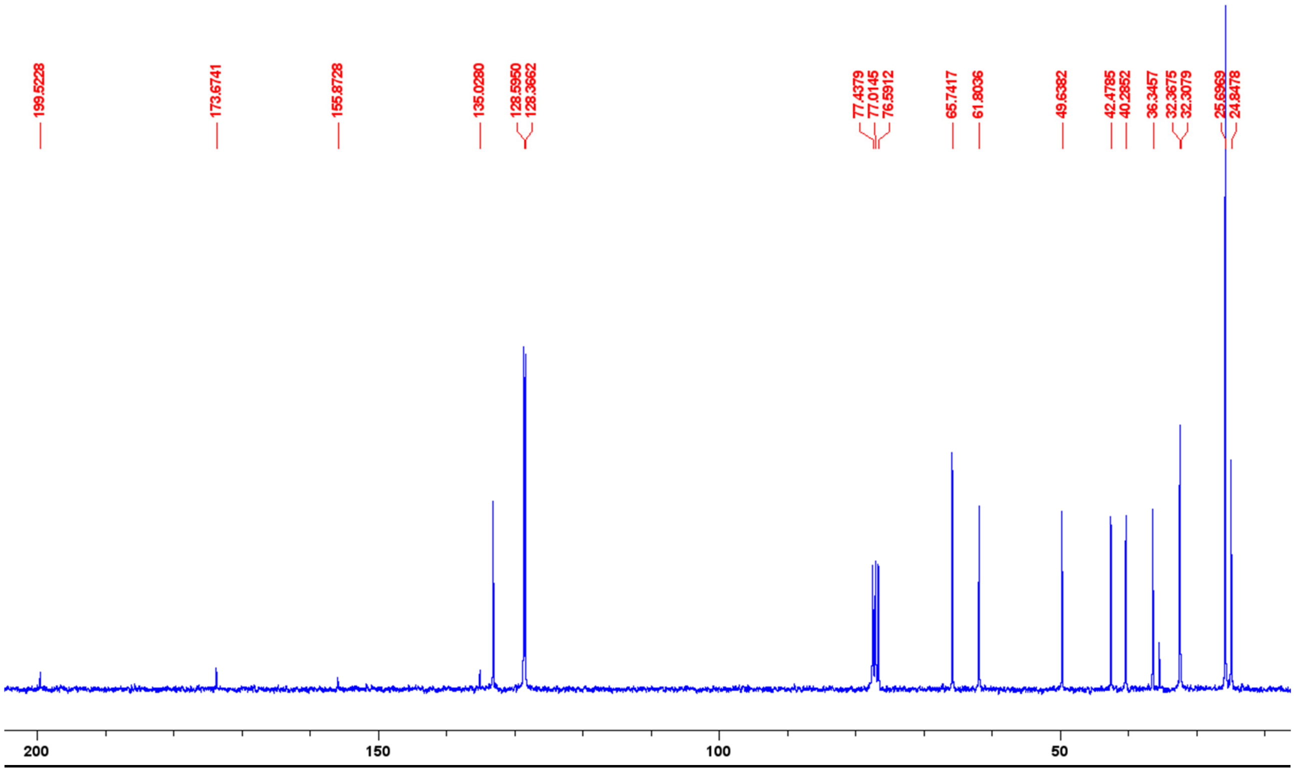

Compound 9b

1H-NMR:

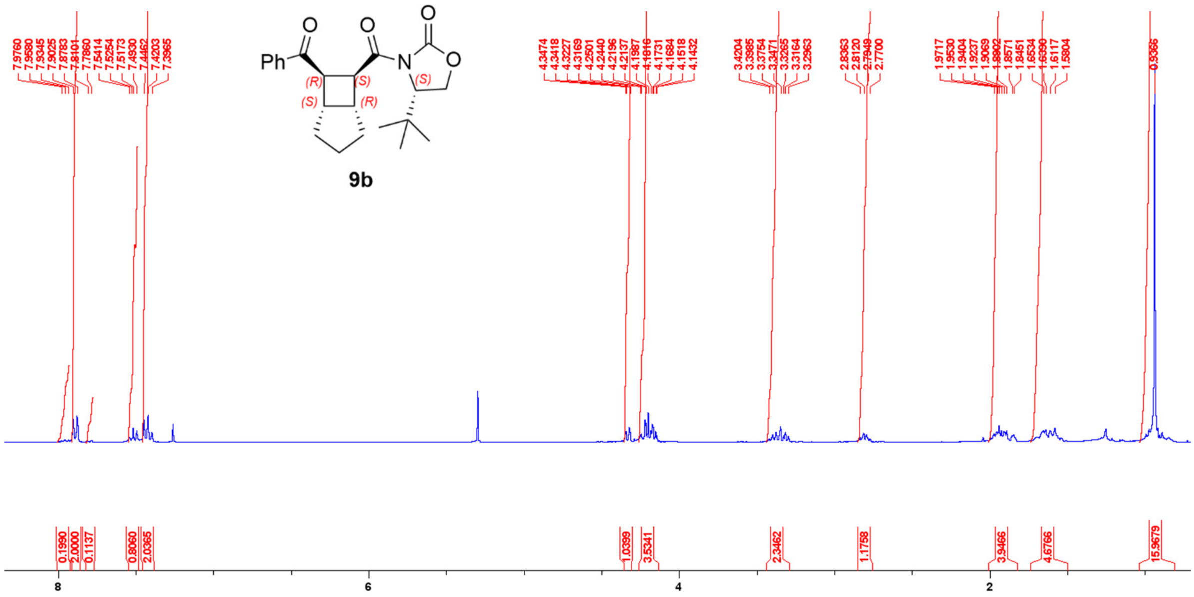

9b

13C-NMR:

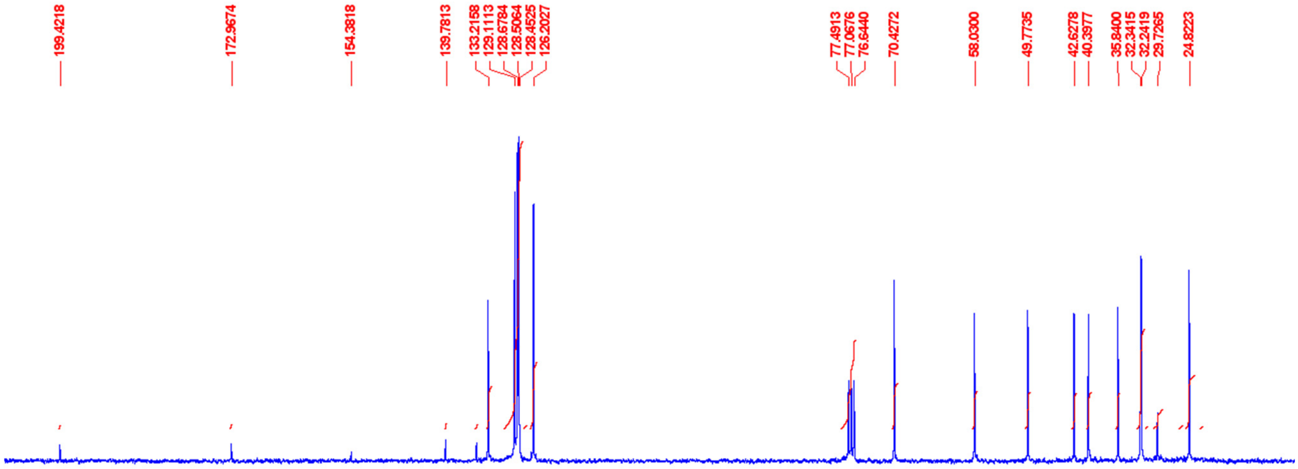

Compound 10a

1H-NMR:

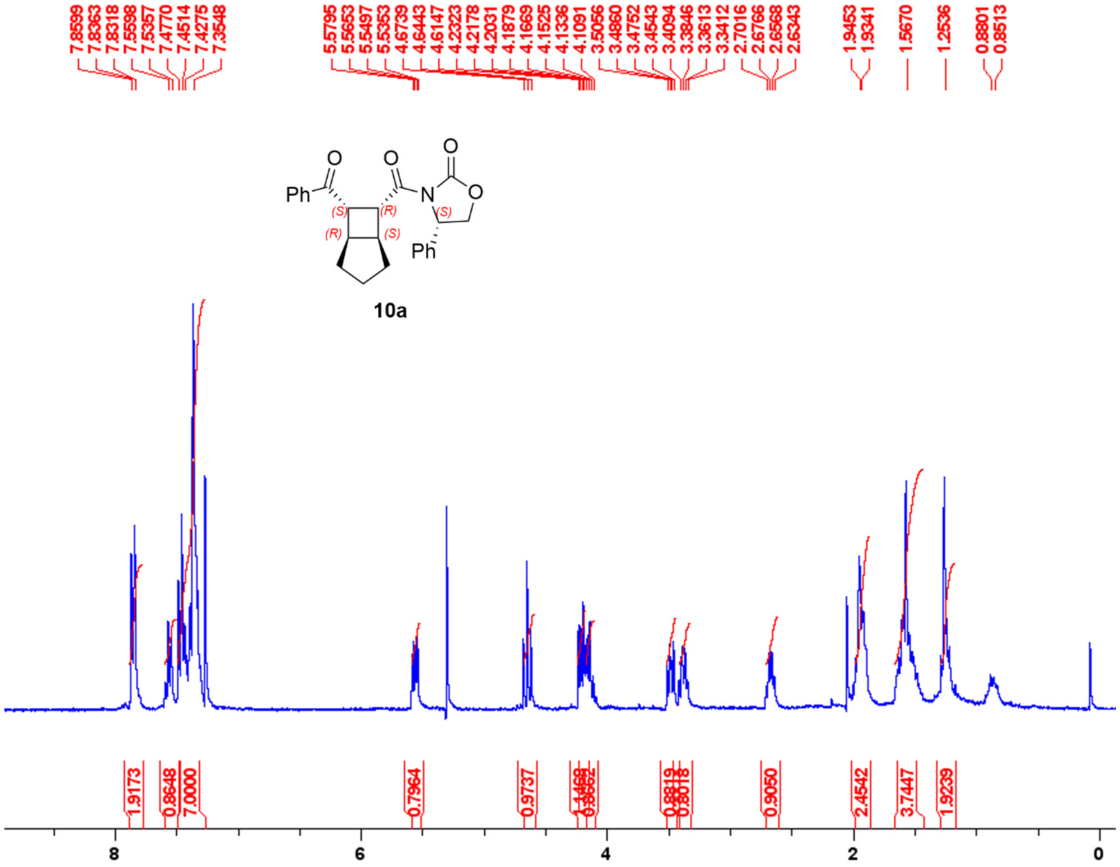

13C-NMR:

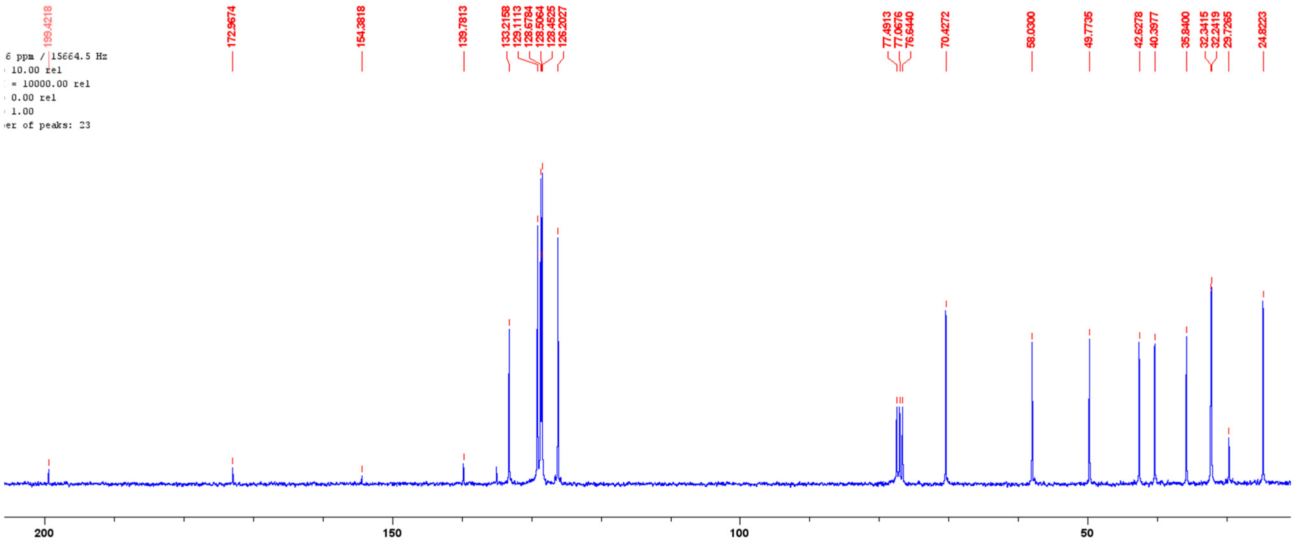

1H-COSY:

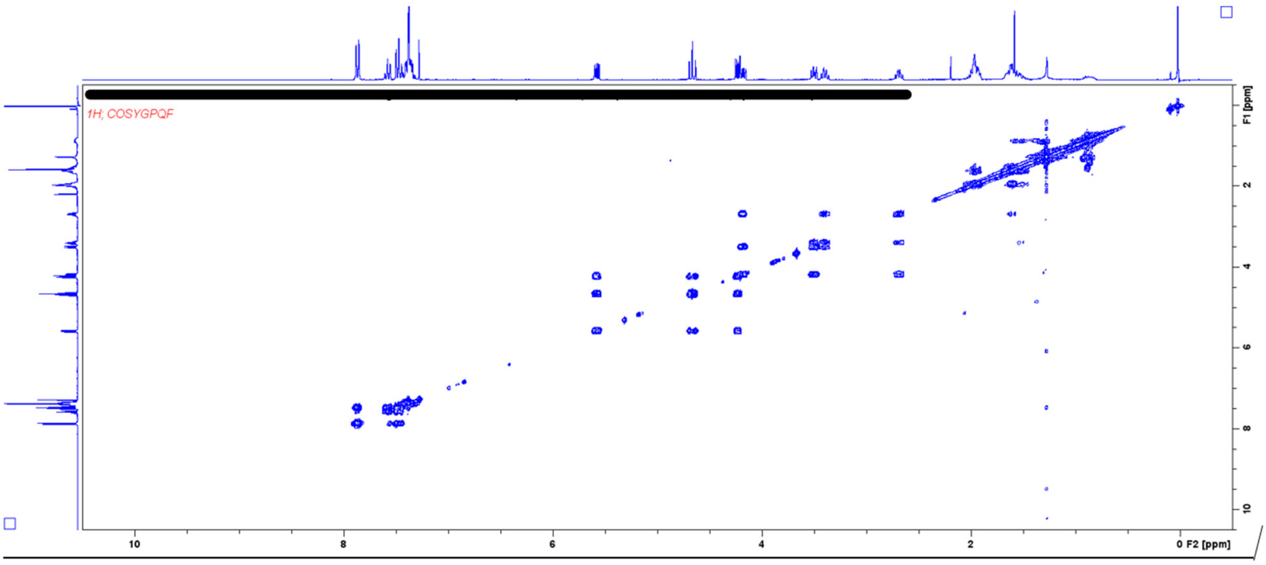

13C-HMBC:

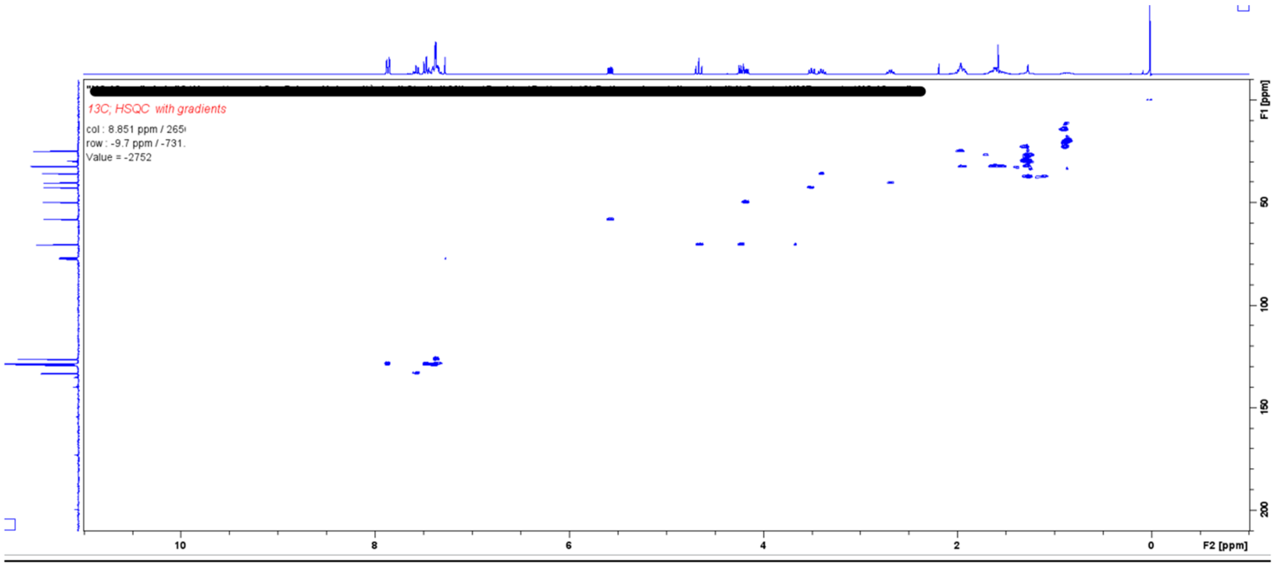

13C-HSQC:

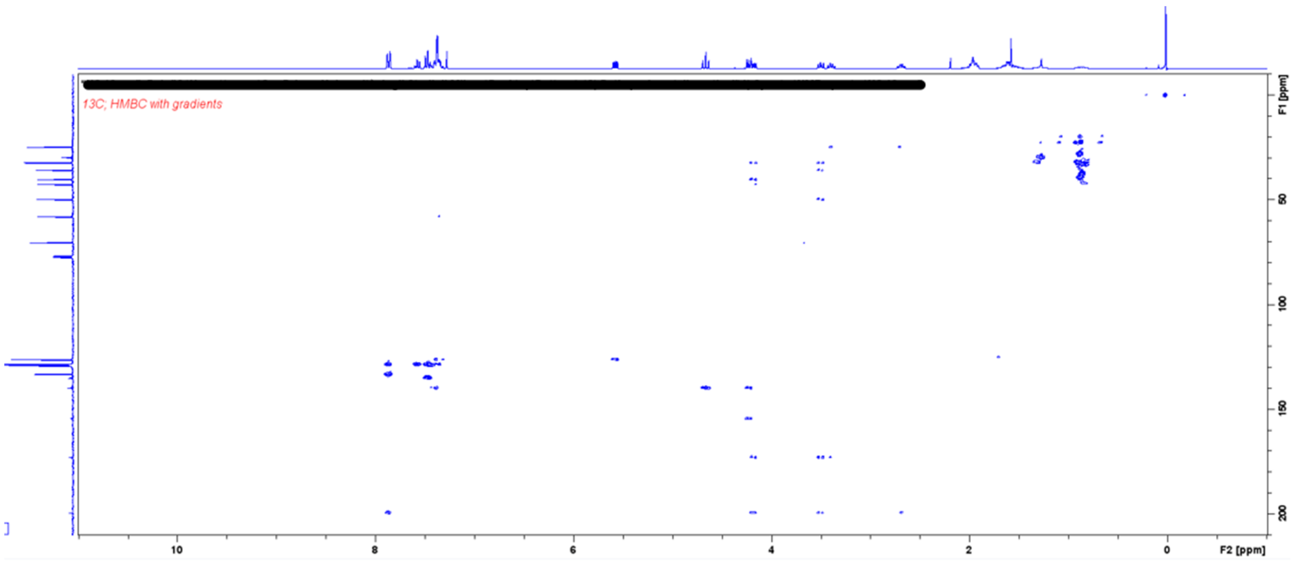

Compound 10b

1H-NMR:

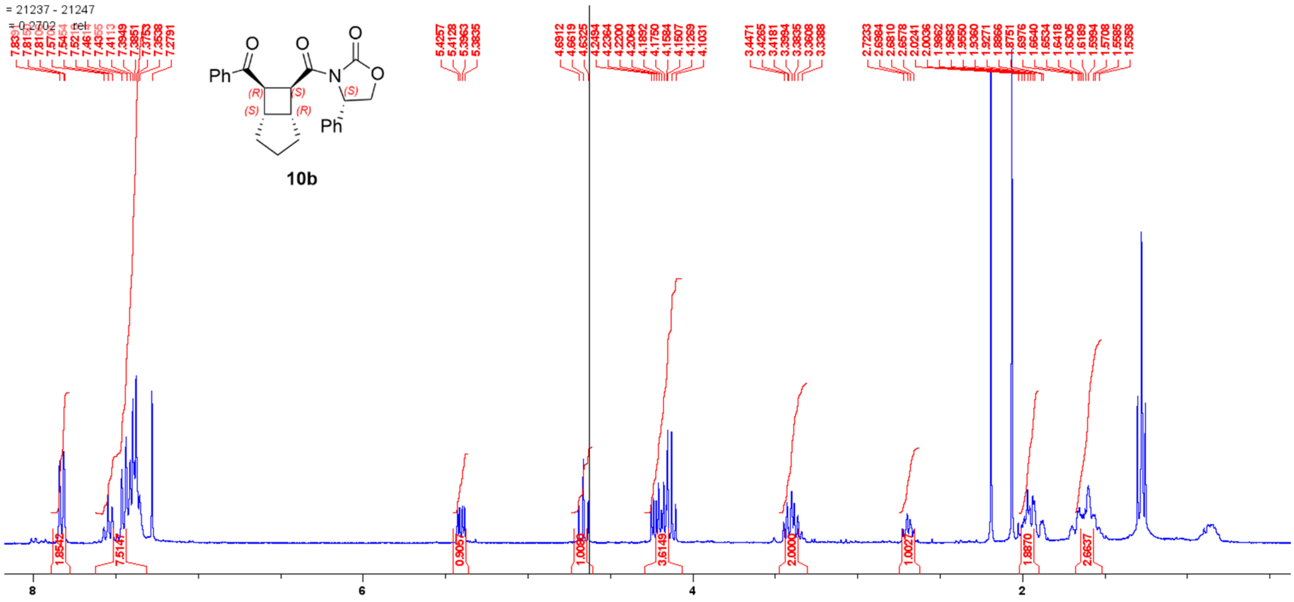

13C-NMR:

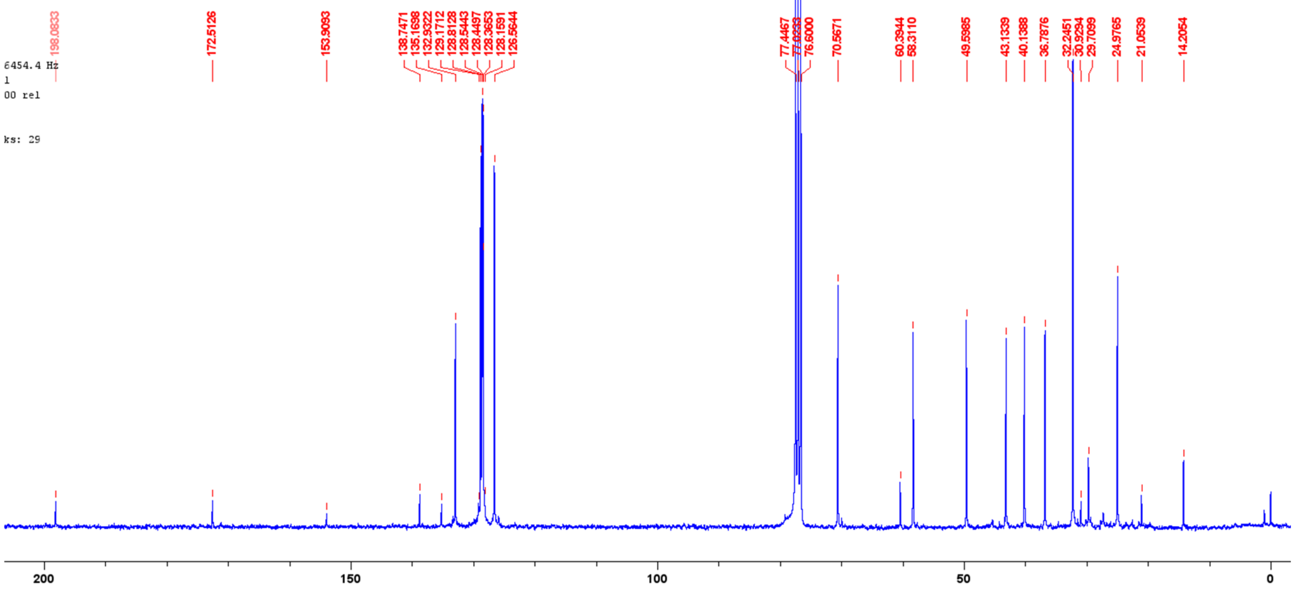

1H-COSY:

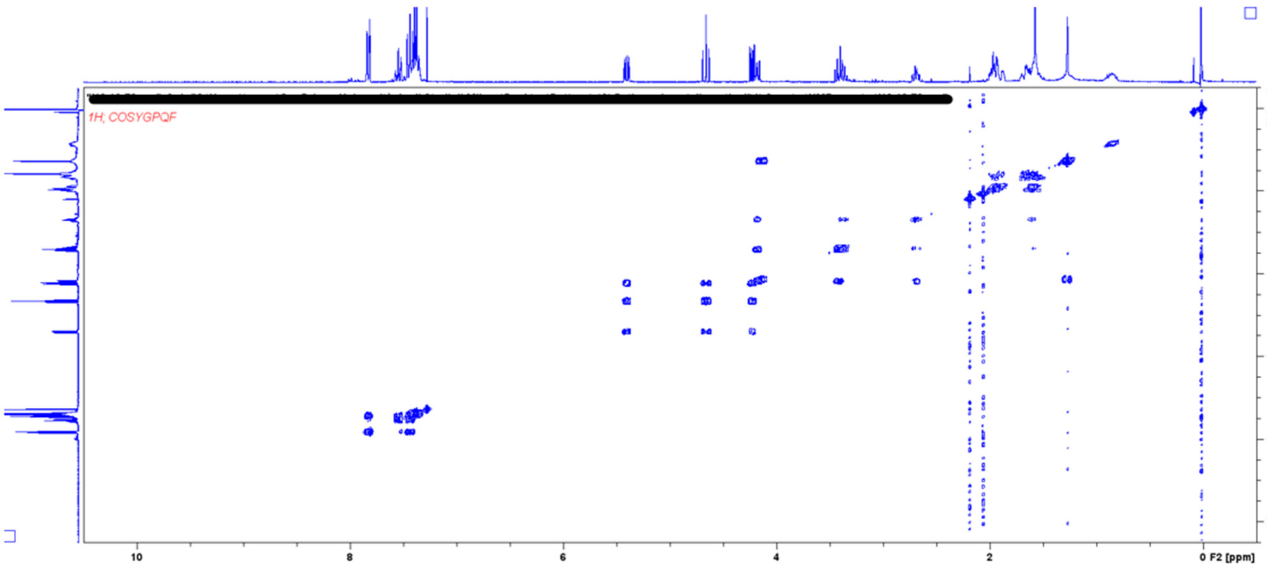

13C-HSQC:

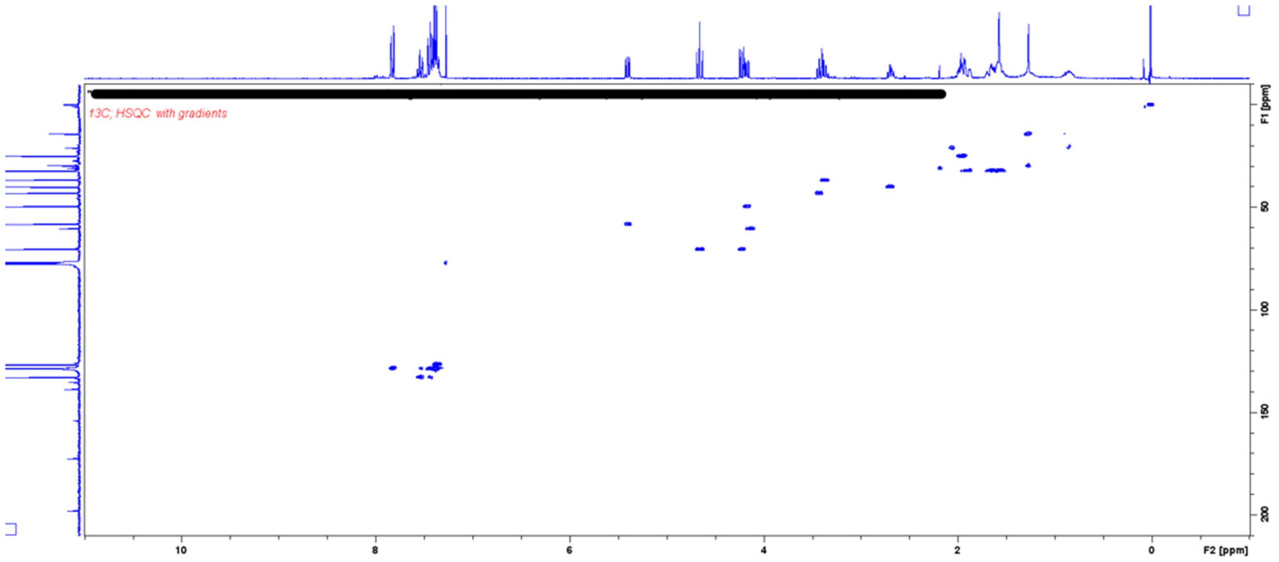

S6. DFT calculations

Preliminary structures corresponding to intermediates (F_{cis}, F_{trans}, Ga, Gb, Gc, Gd, Haa, Hab, Hac, Had, Hba, Hbb, Hbc, Hbd) and compounds Iaa, Iab, Iac, Iad, Iba, Ibb, Ibc and Ibd were generated through Monte Carlo conformational analysis performed with Molecular Mechanics calculations using the MMFFs force field[9] of the Macromodel package in the Schrodinger suite. [10] All geometries of structures obtained within a 3 kcal/mol range were then fully optimized as minima by DFT approach using the unrestricted M062X functional[11] with the 6-31G(d,p) basis set of the Gaussian package, [12] including solvation effects of acetonitrile using the PCM model.[13] Among these structures, the one exhibiting the lowest energy was selected to construct the reaction profile.
Transition states were directly located through a DFT approach, with harmonic vibrational calculations also conducted at the same level of theory, indicating the presence of one imaginary frequency.

Effects of the coordination with LiBr

The effects of coordination between compound **8** and **LiBr** was investigated considering the isodesmic-type reaction showed in scheme S1.

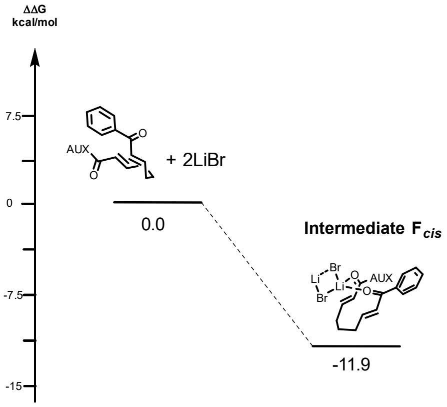

Scheme S1. Effect of coordination with LiBr

Since $\Delta G_{(product\text{-}reactants)} = -11.9$ kcal/mol, the coordinative process is favorite.

Steric hinderance of phenyl group of oxazolidinone auxiliary

During the second ring closure, the relative position of the phenyl group disfavors the formation of compound Hba due to steric hindrance resulting from its proximity to the reaction site (Figure S4).

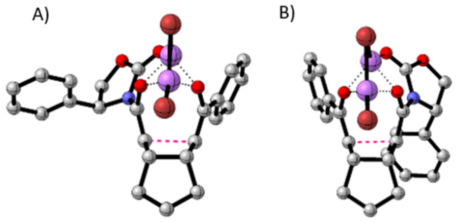

Figure S4. Relative position of the phenyl group for the synthesis of compound **TS2**$_{(Ga\rightarrow Haa)}$ (A) and compound **TS2**$_{(Ga\rightarrow Hab)}$ (B)

Free Gibbs energy obtained for all optimized structures

Relative Gibbs Free Energies G (in kcal/mol) and frequencies analysis of transitions states (TS) computed at the (U)M06-2X/6-31G(d,p)/PCM (CH_3CN) Level are reported in table S1.

#	Compound Name	G (kcal/mol)	Imaginary frequency (cm^{-1})	ΔG (kca/mol)
1	Intermediate F_{cis}	-6442,262825	-	0,0
2	Intermediate F_{trans}	-6442,249485	-	8,4
3	$TS1_{(Fcis\text{-}Ga)}$	-6442,26305	-515,22	-0,1
4	$TS1_{(Fcis\text{-}Gb)}$	-6442,256861	-407,13	3,7
5	$TS1_{(Ftrans\text{-}Gc)}$	-6442,244429	-192,81	11,5
6	$TS1_{(Ftrans\text{ -}Gd)}$	-6442,206362	-152,42	35,4
7	Ga	-6442,272919	-	-6,3
8	Gb	-6442,271211	-	-5,3
9	Gc	-6442,25537	-	4,7
10	Gd	-6442,248171	-	9,2
11	$TS2_{(Ga\text{-}Haa)}$	-6442,266963	-327,26	-2,6
12	$TS2_{(Gb\text{-}Hba)}$	-6442,262351	-214,94	0,3
13	$TS2_{(Ga\text{-}Hab)}$	-6442,231403	-390,83	19,7
14	$TS2_{(Gb\text{-}Hbb)}$	-6442,228621	-383,7	21,5
15	$TS2_{(Ga\text{-}Hac)}$	-6442,236529	-219,89	16,5
16	$TS2_{(Gb\text{-}Hbc)}$	-6442,244034	-256,81	11,8
17	$TS2_{(Ga\text{-}Had)}$	-6442,24003	-233,55	14,3
18	$TS2_{(Gb\text{-}Hbd)}$	-6442,233989	-387,61	18,1
19	Haa	-6442,272636	-	-6,2
20	Hba	-6442,265488	-	-1,7
21	Hab	-6442,235097	-	17,4
22	Hbb	-6442,247509	-	9,6
23	Hac	-6442,252366	-	6,6
24	Hbc	-6442,248984	-	8,7
25	Had	-6442,248047	-	9,3
26	Hbd	-6442,244627	-	11,4
27	Iaa	-6442,163077	-	-55,8
28	Iba	-6442,104556	-	-19,1
29	Iab	-6442,159513	-	-53,6

30	Ibb	-6442,164664	-	-56,8
31	Iac	-6.442,151496	-	-48,5
32	Ibc	-6442,151494	-	-48,5
33	Iad	-6442,171271	-	-61,0
34	Ibd	-6442,176163	-	-64,0
35	LiBr	-2579,46409	-	-
36	EosinY	-11430,78194	-	-
37	EosinY radical anion	-11428,52147	-	-
38	iPr2NEt	-370,63731	-	-
39	iPr2NEt radical cation	-370,44862	-	-
40	Compound 8_LiBr	-6442,152177	-	-
41	Compound 8_noLiBr	-1283,205061	-	11,9

Table S1

Geometries (XYZ coordinates)

Compound 8 w/o LiBr (charge = 0; spin = 1)

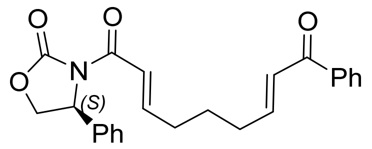

C	-0.63509	3.28981	1.14577
C	0.52222	2.63913	0.72013
C	0.72519	2.38668	-0.64080
C	-0.24523	2.78653	-1.56730
C	-1.40640	3.42064	-1.14061
C	-1.60083	3.67648	0.21860
C	1.96550	1.72387	-1.16472
O	2.26091	1.83680	-2.34593
C	2.78062	0.91202	-0.22889
C	3.88985	0.29608	-0.65145
C	4.73855	-0.62860	0.16727
C	4.85095	-2.03902	-0.44814
C	3.67083	-2.96484	-0.09406
C	2.36375	-2.33501	-0.44655
C	1.42081	-1.99904	0.43805
C	0.26332	-1.20952	-0.02789
O	-0.04498	-1.06178	-1.19636
N	-0.54393	-0.61181	0.95735
C	-1.79850	0.04463	0.57618
C	-2.27151	0.51171	1.96175
O	-1.07582	0.59073	2.75244
C	-0.09350	-0.12506	2.18150
O	0.99602	-0.25795	2.68238
C	-2.78950	-0.86154	-0.11121
C	-3.51367	-0.38029	-1.20031
C	-4.47147	-1.18277	-1.81654
C	-4.70196	-2.47468	-1.35038
C	-3.97489	-2.96094	-0.26408
C	-3.02519	-2.15575	0.35700
H	-0.78225	3.48718	2.20259
H	1.26763	2.35151	1.45536
H	-0.06964	2.58742	-2.61909
H	-2.15889	3.71898	-1.86311
H	-2.50425	4.17619	0.55341
H	2.41704	0.74422	0.78185

H	4.19539	0.45701	-1.68613
H	5.74541	-0.19835	0.22814
H	4.34889	-0.69289	1.19001
H	4.93702	-1.95348	-1.53778
H	5.77101	-2.51387	-0.09687
H	3.79223	-3.90713	-0.64103
H	3.69301	-3.19642	0.97594
H	2.21184	-2.05012	-1.48800
H	1.52295	-2.20557	1.49609
H	-1.57279	0.89863	-0.07111
H	-2.73790	1.49590	1.93994
H	-2.94211	-0.21871	2.42199
H	-3.32076	0.62457	-1.56778
H	-5.03111	-0.80100	-2.66412
H	-5.44312	-3.10310	-1.83317
H	-4.14852	-3.96849	0.09943
H	-2.45581	-2.53535	1.20186

Compound 8 (charge = 0; spin = 1)

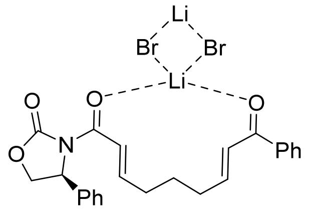

C	-2.63388	-2.51670	3.15976
C	-3.16052	-2.14515	1.92638
C	-2.83722	-0.89813	1.37789
C	-2.01568	-0.01548	2.09227
C	-1.47915	-0.39628	3.31596
C	-1.78322	-1.65193	3.84618
C	-3.33551	-0.44605	0.05113
O	-3.34656	0.76081	-0.21598
C	-3.72612	-1.39859	-1.00221
C	-3.09017	-2.56457	-1.17960
C	-3.15575	-3.37745	-2.43473
C	-2.08411	-2.90146	-3.43979
C	-0.67005	-2.76663	-2.84179
C	-0.46695	-1.49441	-2.07973
C	0.02820	-1.40378	-0.83762
C	0.19776	-0.08545	-0.21645
O	-0.36124	0.93224	-0.64195
N	1.02230	-0.01154	0.89119
C	1.81109	-1.12580	1.45252
C	2.59008	-0.37245	2.54617
O	1.83754	0.82908	2.78405
C	1.02954	1.08257	1.76210
O	0.38315	2.10269	1.67504
C	2.73281	-1.80469	0.46673
C	3.43112	-1.05560	-0.48367
C	4.29910	-1.69664	-1.36213
C	4.47940	-3.07788	-1.29203
C	3.78629	-3.82142	-0.34054
C	2.90982	-3.18566	0.53659
Li	0.31864	2.78831	-0.19915
Br	2.63479	2.69596	-0.79822
Li	-2.09477	1.64485	-1.42029
Br	-1.72497	3.99223	-0.78461
H	-2.88937	-3.48032	3.58637
H	-3.83602	-2.81164	1.40142
H	-1.79915	0.96057	1.66826
H	-0.83348	0.28492	3.86244

H	-1.36748	-1.95008	4.80304
H	-4.38971	-0.99895	-1.76636
H	-2.37784	-2.89435	-0.42619
H	-2.98503	-4.43204	-2.19923
H	-4.14205	-3.28755	-2.89919
H	-2.05368	-3.60785	-4.27304
H	-2.39115	-1.93275	-3.85115
H	-0.42257	-3.63256	-2.21818
H	0.04837	-2.75263	-3.67092
H	-0.71929	-0.57139	-2.60295
H	0.32034	-2.28973	-0.28497
H	1.12442	-1.85090	1.90381
H	2.65670	-0.92824	3.47867
H	3.58332	-0.08333	2.19670
H	3.28783	0.02259	-0.54214
H	4.83480	-1.11527	-2.10535
H	5.15661	-3.57284	-1.98017
H	3.91997	-4.89644	-0.28356
H	2.35858	-3.76540	1.27226

Intermediate F_{cis} LiBr (charge = -1; spin = 2)

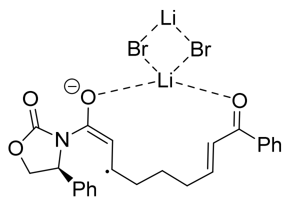

C	3.54405	-1.50164	2.60921
C	2.81721	-2.20875	1.65996
C	1.40584	-2.17371	1.65240
C	0.76614	-1.45931	2.68827
C	1.49864	-0.76302	3.64245
C	2.89371	-0.75910	3.59950
C	0.55557	-2.89753	0.69027
O	-0.69505	-3.00556	0.95166
C	1.05292	-3.54230	-0.51007
C	2.09731	-3.14861	-1.27542
C	2.48751	-3.73542	-2.59935
C	1.94517	-2.89518	-3.77940
C	1.93798	-1.38555	-3.48944
C	0.88668	-1.03905	-2.48368
C	1.02690	-0.24584	-1.39353
C	-0.09539	-0.03610	-0.51850
O	-1.23836	-0.51654	-0.73742
N	0.08379	0.78663	0.60150
C	1.36040	1.40013	0.99336
C	0.93481	2.14576	2.27594
O	-0.34859	1.61408	2.62575
C	-0.84737	0.88815	1.62131
O	-1.96791	0.41845	1.67342
C	1.94656	2.35424	-0.02280
C	1.12216	3.18276	-0.78771
C	1.69133	4.08685	-1.67985
C	3.07774	4.17449	-1.80734
C	3.89803	3.35046	-1.04137
C	3.33207	2.43830	-0.15227
Li	-2.84365	0.42374	-0.11630
Br	-2.75351	2.76807	-0.63805
Li	-1.99171	-2.21588	-0.09029
Br	-4.36137	-1.57934	-0.13467
H	4.62879	-1.54353	2.58874
H	3.34728	-2.81863	0.93848

H	-0.31810	-1.46975	2.72317
H	0.97594	-0.21376	4.42080
H	3.46601	-0.20817	4.33872
H	0.39403	-4.32457	-0.89109
H	2.70278	-2.31125	-0.94273
H	3.58093	-3.77129	-2.66510
H	2.12167	-4.76326	-2.68962
H	2.52563	-3.10334	-4.68344
H	0.91316	-3.20375	-3.98489
H	2.92429	-1.03917	-3.15983
H	1.70843	-0.85939	-4.42619
H	-0.10696	-1.44056	-2.68109
H	1.99351	0.17220	-1.13881
H	2.07770	0.60392	1.22968
H	1.62198	1.97095	3.10303
H	0.81701	3.21590	2.09369
H	0.03958	3.10954	-0.69311
H	1.04978	4.72410	-2.28004
H	3.51617	4.88042	-2.50511
H	4.97699	3.41081	-1.13888
H	3.96741	1.78342	0.43923

Intermediate F_{trans} LiBr (charge = -1; spin = 2)

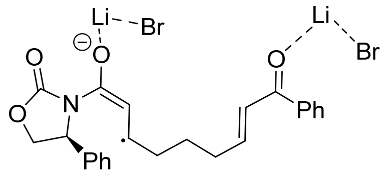

C	-3.32172	2.95245	1.94758
C	-2.31145	2.02846	2.66753
C	-1.93143	0.81911	1.86590
C	-1.60985	3.19721	0.08844
C	-3.06525	3.15089	0.43687
C	-1.07124	2.56913	-0.96963
C	0.36466	2.33559	-1.10877
O	0.76312	1.36776	-1.77177
C	1.35265	3.21863	-0.42185
C	1.21144	4.61007	-0.44564
C	2.18118	5.41693	0.14300
C	3.28012	4.83741	0.77445
C	3.41445	3.44948	0.81230
C	2.46037	2.63860	0.20685
C	-0.61201	0.51747	1.58810
C	0.00873	-0.40459	0.73182
O	1.29198	-0.41834	0.53671
N	-0.72486	-1.38809	-0.00670
C	-2.04413	-1.93201	0.32044
C	-1.86294	-3.36536	-0.20124
O	-0.91984	-3.20281	-1.27527
C	-0.16722	-2.12139	-1.00898
O	0.85105	-1.88546	-1.65846
Li	1.80752	-0.18196	-1.33476
Br	4.15169	-0.89931	-0.95135
C	-3.17749	-1.21223	-0.39078
C	-4.47522	-1.32628	0.11424
C	-5.54067	-0.70581	-0.53099
C	-5.31947	0.02993	-1.69487
C	-4.02912	0.13814	-2.20741
C	-2.96105	-0.48033	-1.55778
Li	2.31892	-2.01163	0.36342
Br	1.35025	-3.87661	1.48638
H	-4.33246	2.54574	2.05890

H	-3.32294	3.92380	2.45401
H	-1.40261	2.59499	2.90421
H	-2.75678	1.75505	3.63552
H	-2.75272	0.25662	1.43711
H	-0.95315	3.72209	0.78239
H	-3.53304	2.34565	-0.14045
H	-3.54610	4.08706	0.12369
H	-1.69976	1.98433	-1.63666
H	0.35530	5.05579	-0.94260
H	2.07878	6.49636	0.10864
H	4.03155	5.46767	1.23901
H	4.26420	2.99794	1.31404
H	2.54175	1.55614	0.25405
H	0.13939	1.16514	2.04178
H	-2.19429	-1.92353	1.40249
H	-1.40382	-4.00907	0.55253
H	-2.77573	-3.79938	-0.60522
H	-4.64778	-1.89204	1.02676
H	-6.54248	-0.79212	-0.12309
H	-6.14856	0.51714	-2.19727
H	-3.84880	0.70966	-3.11266
H	-1.95355	-0.36859	-1.94947

TS1($_{Fcis\text{-}Ga}$) LiBr (charge = -1; spin = 2)

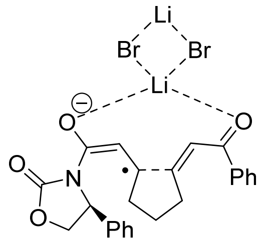

C	0.64140	-0.26140	-0.38240
C	0.80620	-1.16760	0.63990
C	-0.26110	-2.01970	1.08560
C	0.09610	-3.25500	1.89120
C	-1.51630	-1.24500	2.35260
C	-1.04580	0.04590	2.65610
C	-1.17320	1.12020	1.73340
O	-0.39580	-0.08310	-1.11330
C	-1.52670	-2.32500	3.41750
C	-1.11380	-3.63230	2.73930
Li	-1.11900	1.76110	-1.04230
Br	-3.62470	-2.30580	-0.55320
Br	-3.12240	1.42160	-2.38110
Li	-2.30430	-0.30880	-0.67860
C	-0.29180	2.31560	1.86410
O	-1.96470	1.08640	0.74320
C	-0.76260	3.56990	1.46230
C	0.08690	4.67090	1.46550
C	1.42130	4.52160	1.84220
C	1.90110	3.27160	2.23210
C	1.04530	2.17390	2.25490
H	1.73040	-1.16300	1.20850
H	-1.01800	-2.21830	0.32420
H	0.95640	-3.03740	2.53660
H	0.39190	-4.05950	1.20810
H	-2.37030	-1.28250	1.67490
H	-0.38840	0.18950	3.51010
H	-2.51460	-2.39800	3.88390
H	-0.80600	-2.07340	4.20510
H	-0.89600	-4.42740	3.45700

H	-1.92360	-3.97750	2.08490
H	-1.79470	3.66680	1.13700
H	-0.28710	5.64400	1.16450
H	2.08800	5.37770	1.82830
H	2.94260	3.15060	2.51280
H	1.42080	1.19330	2.53630
C	3.14140	0.32610	-0.26340
C	3.85300	1.51240	-0.93450
H	4.64990	1.93600	-0.32610
H	4.23260	1.23710	-1.92220
N	1.76530	0.59040	-0.68920
C	1.62550	1.90600	-1.02920
O	0.60220	2.53880	-1.23840
O	2.82620	2.50020	-1.10100
C	3.68270	-1.00920	-0.71410
C	3.38870	-1.49950	-1.98870
C	4.52350	-1.73570	0.12660
C	3.93040	-2.70790	-2.41370
H	2.72610	-0.93590	-2.64060
C	5.07240	-2.94310	-0.30230
H	4.74600	-1.35820	1.12130
C	4.77570	-3.43100	-1.57190
H	3.69230	-3.08730	-3.40200
H	5.72580	-3.50340	0.35840
H	5.19740	-4.37370	-1.90480
H	3.21530	0.40990	0.82920

TS1($_{Fcis\text{-}Ga}$) LiBr (charge = -1; spin = 2)

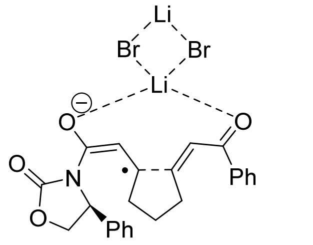

C	0.04045	0.50248	-1.38210
C	0.30363	1.70260	-0.75213
C	-0.72587	2.48473	-0.13784
C	-0.42621	3.96842	0.09566
C	-1.20394	1.99673	1.67970
C	-0.30334	1.04945	2.21348
C	-0.35671	-0.31629	1.84985
O	-1.08083	-0.09291	-1.50563
C	-1.21314	3.38604	2.30144
C	-0.13864	4.19419	1.57936
Li	-0.95943	-1.88545	-0.56475
Br	-4.37859	1.46773	-0.19952
Br	-3.28692	-2.52440	-0.90068
Li	-2.58823	-0.12663	-0.26822
C	0.64808	-1.28327	2.38510
O	-1.26314	-0.77255	1.08390
C	0.32636	-2.64727	2.40393
C	1.23307	-3.58235	2.89173
C	2.47686	-3.16818	3.36603
C	2.80828	-1.81442	3.34758
C	1.90180	-0.87703	2.86116
H	1.33498	2.02692	-0.65853
H	-1.72073	2.28447	-0.53832
H	0.40813	4.28919	-0.53427
H	-1.30438	4.55693	-0.19146
H	-2.18023	1.59380	1.41203

H	0.49867	1.38783	2.86311
H	-2.19137	3.85256	2.14047
H	-1.04107	3.33667	3.38098
H	0.85100	3.78931	1.82729
H	-0.14568	5.25337	1.85080
H	-0.64633	-2.96419	2.03961
H	0.96717	-4.63421	2.90784
H	3.18499	-3.89623	3.74771
H	3.77697	-1.48767	3.71286
H	2.18704	0.16969	2.82599
C	2.36215	0.48488	-2.51573
C	2.82057	-0.60747	-3.49954
H	3.90261	-0.70108	-3.56778
H	2.37857	-0.47576	-4.48959
N	1.15113	-0.15752	-2.01270
C	1.24798	-1.50994	-2.14859
O	0.55622	-2.37722	-1.63330
O	2.29629	-1.82006	-2.92548
C	3.40738	0.76175	-1.44884
C	3.41268	0.09222	-0.22467
C	4.43049	1.66612	-1.74216
C	4.44135	0.31811	0.68822
H	2.60577	-0.59112	0.03274
C	5.45483	1.89307	-0.82804
H	4.42384	2.19426	-2.69244
C	5.46396	1.21487	0.38942
H	4.44100	-0.21195	1.63511
H	6.24292	2.60021	-1.06495
H	6.26168	1.38848	1.10396
H	2.10170	1.40583	-3.04257

TS1($_{Ftrans\text{-}Gc}$) LiBr (charge = -1; spin = 2)

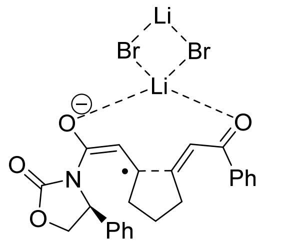

C	-3.78366	2.38542	1.84817
C	-2.60628	1.65036	2.52726
C	-1.81884	0.93341	1.45925
C	-1.93162	2.52985	0.19616
C	-3.43062	2.61014	0.35860
C	-1.30029	2.14795	-0.99096
C	0.11027	2.18654	-1.15669
O	0.71284	1.39588	-1.94515
C	0.93675	3.18719	-0.40164
C	0.51328	4.51606	-0.27341
C	1.31641	5.45830	0.36290
C	2.55188	5.08224	0.88657
C	2.98372	3.76270	0.75912
C	2.18794	2.82336	0.11010

C	-0.48415	0.52076	1.60468
C	0.18655	-0.31392	0.70196
O	1.44428	-0.29400	0.52604
N	-0.53751	-1.27414	-0.07957
C	-1.77387	-1.96515	0.32635
C	-1.43869	-3.39816	-0.11565
O	-0.53237	-3.19556	-1.21553
C	0.09477	-2.02615	-1.04918
O	1.05094	-1.71611	-1.74131
Li	1.90491	0.03130	-1.43441
Br	4.29437	-0.43422	-1.00410
C	-3.01908	-1.45342	-0.37674
C	-4.24663	-1.50073	0.28808
C	-5.40849	-1.06479	-0.34313
C	-5.35351	-0.58279	-1.65010
C	-4.13260	-0.54075	-2.32004
C	-2.96947	-0.97501	-1.68686
Li	2.72062	-1.70065	0.51197
Br	1.70731	-3.55721	1.62254
H	-4.70100	1.79553	1.92739
H	-3.97503	3.33674	2.35096
H	-1.95119	2.35042	3.05760
H	-2.96902	0.93111	3.27088
H	-2.44771	0.32595	0.81922
H	-1.37036	3.19712	0.84998
H	-3.89519	1.84153	-0.27271
H	-3.80386	3.57889	0.00834
H	-1.84489	1.52161	-1.69556
H	-0.44428	4.81030	-0.69256
H	0.98000	6.48687	0.44449
H	3.17760	5.81485	1.38624
H	3.94524	3.46280	1.16432
H	2.53323	1.79856	0.01986
H	0.17935	1.07997	2.25940
H	-1.88670	-1.90031	1.41093
H	-0.90398	-3.94862	0.66020
H	-2.30321	-3.94766	-0.48292
H	-4.28766	-1.86243	1.31257
H	-6.35425	-1.09512	0.18795
H	-6.25691	-0.23803	-2.14214
H	-4.08165	-0.16369	-3.33635
H	-2.01942	-0.91134	-2.21063

TS1($_{\textit{Ftrans}\text{-Gd}}$) LiBr (charge = -1; spin = 2)

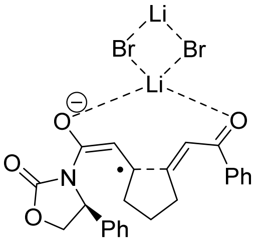

C	1.36252	-0.99497	-1.43543
C	2.76494	-0.86081	-1.50572
C	0.54003	-1.90149	-0.53948
C	-0.50547	1.00607	-0.95967
C	0.75609	0.69556	-0.39670
C	1.03010	0.03719	0.94450
H	1.55347	1.34602	-0.74775
H	0.56612	0.56815	1.77950
H	2.11109	0.10475	1.10788
H	-0.48749	1.47574	-1.94030

O	-2.79037	0.52513	-1.33440
H	0.87316	-0.74601	-2.37436
C	0.62618	-1.45331	0.93197
H	0.81173	-2.95315	-0.65438
H	-0.49912	-1.80847	-0.87391
H	-0.33714	-1.61461	1.42176
H	1.36333	-2.03898	1.48688
C	3.79852	-1.21991	-0.60418
H	3.11704	-0.19446	-2.29381
O	4.91690	-0.61994	-0.61341
C	3.46932	-4.42707	2.22024
C	3.98912	-3.19223	2.61123
C	4.10086	-2.16106	1.68499
C	3.66240	-2.34454	0.36900
C	3.17010	-3.59106	-0.02364
C	3.07318	-4.62947	0.89957
H	3.38482	-5.23322	2.94184
H	4.30899	-3.03741	3.63662
H	4.51022	-1.19741	1.97359
H	2.87560	-3.74351	-1.05782
H	2.69276	-5.59669	0.58749
Li	5.35611	1.13950	-1.05201
Li	-3.95690	-0.95962	-1.21232
Br	7.03032	2.82665	-1.15780
Br	-5.78160	-1.93890	-2.33075
C	-1.78557	0.58829	-0.57932
C	-1.97889	1.37218	1.83032
C	-2.37436	0.54593	3.06178
H	-0.97255	1.77911	1.93350
H	-1.49863	0.13718	3.57104
H	-3.00554	1.08714	3.76478
N	-1.99360	0.29256	0.82158
C	-2.85758	-0.68101	1.21832
O	-3.33391	-1.57807	0.53612
O	-3.13681	-0.55439	2.52454
C	-2.93527	2.48228	1.44918
C	-2.43179	3.65728	0.89169
C	-4.31837	2.32268	1.58357
C	-3.29737	4.66906	0.47964
H	-1.35765	3.77689	0.77760
C	-5.18263	3.33266	1.17282
H	-4.72463	1.40931	2.01205
C	-4.67292	4.50772	0.62028
H	-2.89629	5.58118	0.05027
H	-6.25406	3.20361	1.28482
H	-5.34840	5.29471	0.30150

Intermediate Ga LiBr (charge = -1; spin = 2)

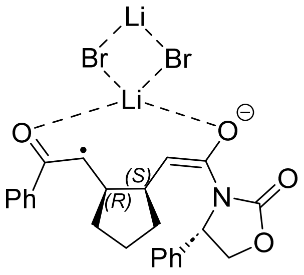

C	0.65472	-0.18592	-0.37861
C	0.85069	-1.06211	0.63963
C	-0.19114	-2.05406	1.07226
C	0.39547	-3.35653	1.65029
C	-1.16626	-1.52238	2.22050

C	-0.79496	-0.13265	2.57736
C	-1.11770	0.96679	1.72304
O	-0.40111	-0.05004	-1.11744
C	-1.05489	-2.54523	3.36297
C	-0.64737	-3.84212	2.65786
Li	-1.17511	1.72773	-1.10391
Br	-3.59248	-2.44025	-0.49597
Br	-3.27511	1.32548	-2.25812
Li	-2.25751	-0.45800	-0.69405
C	-0.38773	2.26019	1.86342
O	-1.97919	0.86912	0.81428
C	-1.03738	3.44875	1.51812
C	-0.34496	4.65452	1.54053
C	1.00649	4.67560	1.88337
C	1.66234	3.49098	2.21709
C	0.96542	2.28634	2.21739
H	1.79275	-1.05465	1.17901
H	-0.82944	-2.30341	0.21744
H	1.33272	-3.13420	2.17725
H	0.62297	-4.08446	0.86655
H	-2.18123	-1.53295	1.80619
H	-0.15138	0.05306	3.43245
H	-1.98007	-2.63162	3.93853
H	-0.25852	-2.24514	4.05621
H	-0.26749	-4.60328	3.34420
H	-1.51037	-4.26365	2.12834
H	-2.08218	3.41300	1.22279
H	-0.85523	5.57665	1.28260
H	1.55030	5.61459	1.88597
H	2.71755	3.50450	2.47005
H	1.47817	1.35742	2.45251
C	3.13317	0.50334	-0.30085
C	3.78919	1.71549	-0.98125
H	4.58593	2.16317	-0.39003
H	4.15475	1.45679	-1.97894
N	1.74223	0.71939	-0.69972
C	1.54817	2.02502	-1.02872
O	0.49793	2.62092	-1.22308
O	2.72524	2.66807	-1.11908
C	3.71192	-0.81318	-0.75993
C	3.41698	-1.31377	-2.03021
C	4.58343	-1.51442	0.07094
C	3.98868	-2.50638	-2.46075
H	2.72873	-0.77113	-2.67331
C	5.16215	-2.70603	-0.36304
H	4.80626	-1.12947	1.06273
C	4.86469	-3.20384	-1.62853
H	3.74973	-2.89412	-3.44563
H	5.83929	-3.24611	0.29050
H	5.30942	-4.13439	-1.96561
H	3.22557	0.58656	0.79077

Intermediate Gb LiBr (charge = -1; spin = 2)

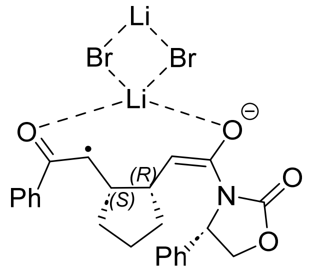

C	0.18730	0.30391	-1.13631
C	0.39597	1.50692	-0.54855
C	-0.72744	2.37044	-0.05233
C	-0.42848	3.87980	-0.12191
C	-1.13038	2.12553	1.47409
C	-0.25887	1.08868	2.07294
C	-0.44474	-0.29969	1.78581
O	-0.94009	-0.30581	-1.33759
C	-1.05546	3.50583	2.14939
C	-1.26652	4.49473	0.99937
Li	-1.01676	-2.03435	-0.47204
Br	-4.37337	1.47845	-0.33416
Br	-3.39314	-2.56874	-0.79030
Li	-2.61320	-0.15142	-0.35424
C	0.61208	-1.28665	2.14771
O	-1.48497	-0.72002	1.22186
C	0.23481	-2.60165	2.44228
C	1.20148	-3.55653	2.73596
C	2.55210	-3.20772	2.71912
C	2.93235	-1.90288	2.41011
C	1.96639	-0.94068	2.12928
H	1.41315	1.85344	-0.40267
H	-1.62822	2.16270	-0.64011
H	0.63656	4.05237	0.08296
H	-0.64602	4.29280	-1.11095
H	-2.16618	1.76551	1.47232
H	0.60283	1.38194	2.66516
H	-1.78222	3.61388	2.95858
H	-0.05640	3.65769	2.57753
H	-0.97279	5.51656	1.25294
H	-2.32449	4.50802	0.71066
H	-0.81950	-2.86292	2.43404
H	0.90458	-4.57207	2.97593
H	3.30786	-3.95368	2.94219
H	3.98404	-1.63533	2.38380
H	2.26863	0.06673	1.85607
C	2.50628	0.18679	-2.29505
C	3.05710	-1.06055	-3.01606
H	4.14436	-1.11643	-3.00404
H	2.68218	-1.13751	-4.03880
N	1.34933	-0.41566	-1.62537
C	1.46338	-1.76027	-1.56567
O	0.75202	-2.56205	-0.96902
O	2.54026	-2.17237	-2.26166
C	3.50921	0.81548	-1.34725
C	4.12776	0.04153	-0.36129
C	3.83732	2.16545	-1.46298
C	5.05367	0.61694	0.50364
H	3.88393	-1.01491	-0.26849
C	4.76350	2.74376	-0.59611
H	3.35594	2.76947	-2.22777
C	5.37054	1.97087	0.38973
H	5.53536	0.01031	1.26419
H	5.00746	3.79672	-0.69105
H	6.09110	2.41861	1.06611
H	2.15618	0.93501	-3.01019

Intermediate Gc LiBr (charge = -1; spin = 2)

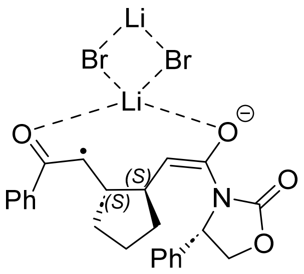

C	3.99562	2.15055	-1.83099
C	2.80602	1.34700	-2.41193
C	1.91757	1.03547	-1.20390
C	2.04502	2.37208	-0.38168
C	3.55714	2.60868	-0.41260
C	1.40170	2.16521	0.93465
C	-0.03290	2.23897	1.10891
O	-0.60794	1.43249	1.86721
C	-0.82444	3.27535	0.38010
C	-0.31867	4.57175	0.24047
C	-1.08335	5.55941	-0.37325
C	-2.35111	5.25197	-0.86337
C	-2.85593	3.95835	-0.73198
C	-2.10161	2.97324	-0.10320
C	0.49859	0.63040	-1.41360
C	-0.20568	-0.23677	-0.64139
O	-1.49505	-0.24466	-0.50643
N	0.47547	-1.25770	0.14932
C	1.65376	-2.02424	-0.28082
C	1.25398	-3.43316	0.18766
O	0.37407	-3.16915	1.29508
C	-0.18690	-1.96126	1.11752
O	-1.12949	-1.60348	1.81236
Li	-1.97988	0.10574	1.35747
Br	-4.37194	-0.34727	0.94380
C	2.95076	-1.58735	0.37965
C	4.15254	-1.71753	-0.31871
C	5.35536	-1.33166	0.26810
C	5.36669	-0.81779	1.56363
C	4.17083	-0.69933	2.27016
C	2.96803	-1.08519	1.68234
Li	-2.74034	-1.63114	-0.53148
Br	-1.83930	-3.54146	-1.64206
H	4.89503	1.53230	-1.76853
H	4.23929	3.00647	-2.46502
H	2.24026	1.95124	-3.13087
H	3.12146	0.43412	-2.92626
H	2.44035	0.29719	-0.59776
H	1.52058	3.14625	-0.95500
H	4.02679	1.97642	0.35173
H	3.82896	3.64491	-0.19760
H	1.94249	1.59947	1.69100
H	0.66824	4.80850	0.62958
H	-0.69108	6.56641	-0.46743
H	-2.94561	6.01966	-1.34796
H	-3.83969	3.71580	-1.12056
H	-2.48734	1.96163	-0.02101
H	-0.12197	1.29496	-2.01189
H	1.74161	-1.97005	-1.36937
H	0.68464	-3.96908	-0.57357
H	2.09579	-4.01934	0.55224
H	4.14219	-2.10206	-1.33565

H	6.28155	-1.42424	-0.28989
H	6.30175	-0.50982	2.01969
H	4.17249	-0.30057	3.27958
H	2.03736	-0.96145	2.23003

Intermediate Gd LiBr (charge = -1; spin = 2)

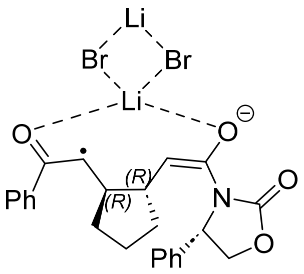

C	4.48456	0.35097	2.16024
C	3.01166	0.40560	2.63162
C	2.19950	0.29491	1.33803
C	3.04747	1.22383	0.39019
C	4.47462	0.73623	0.65259
C	2.50448	1.13615	-0.98568
C	1.28319	1.83647	-1.34191
O	0.40133	1.27294	-2.01940
C	1.09632	3.24592	-0.88370
C	2.15642	4.15097	-0.99053
C	1.97391	5.48391	-0.63381
C	0.73888	5.91040	-0.14956
C	-0.31378	5.00423	-0.02056
C	-0.14111	3.67581	-0.39503
C	0.76174	0.68333	1.32394
C	-0.24427	0.06441	0.66646
O	-1.40179	0.60546	0.38990
N	-0.11195	-1.31555	0.16289
C	0.73377	-1.83569	-0.92746
C	-0.21779	-2.89466	-1.52194
O	-1.07466	-3.23928	-0.42219
C	-1.14527	-2.16970	0.39138
O	-2.04102	-2.04234	1.21633
Li	-1.52590	0.90716	-1.51542
Br	-3.21227	-0.87610	-1.95422
C	2.03033	-2.47974	-0.47269
C	3.14365	-2.44540	-1.31266
C	4.33767	-3.05628	-0.93077
C	4.42281	-3.71281	0.29417
C	3.30548	-3.76948	1.12774
C	2.11399	-3.16220	0.74386
Li	-2.99574	-0.40289	0.55568
Br	-4.90852	0.17115	1.81834
H	5.11520	1.02549	2.74382
H	4.88999	-0.65670	2.28606
H	2.76409	-0.38958	3.33991
H	2.79170	1.36419	3.11645
H	2.31955	-0.72219	0.96263
H	2.91881	2.24376	0.77598
H	5.22488	1.48990	0.40239
H	4.67096	-0.14663	0.03287
H	2.79692	0.30205	-1.61829
H	3.11767	3.81040	-1.36664
H	2.79409	6.18704	-0.73152
H	0.59827	6.94777	0.13588
H	-1.26801	5.33286	0.37751
H	-0.93991	2.95461	-0.24833

H	0.52997	1.69965	1.63950
H	0.92512	-1.04479	-1.65652
H	-0.84601	-2.47767	-2.31283
H	0.29881	-3.79307	-1.85515
H	3.07978	-1.93696	-2.27212
H	5.20022	-3.01209	-1.58776
H	5.35318	-4.18081	0.59830
H	3.36444	-4.28411	2.08130
H	1.25627	-3.18120	1.41063

TS2 ($_{Ga-Haa}$) (charge = -1; spin = 2)

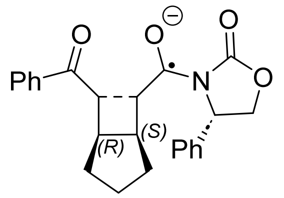

C	-0.97157	-0.97112	0.77926
C	-0.96727	-1.61572	-0.48735
C	0.08654	-2.67935	-0.72618
C	-0.09107	-3.53688	-1.98453
C	1.27247	-1.74516	-1.08017
C	0.46372	-0.49087	-1.40089
C	0.68483	0.76232	-0.78260
O	-0.18212	-1.24405	1.70419
C	2.01518	-2.38220	-2.27652
C	1.35226	-3.76246	-2.45087
Li	0.97981	0.25059	2.11404
Br	4.85585	0.18459	-1.10805
Br	3.24361	-0.39818	2.82346
Li	3.20466	0.53607	0.52265
C	-0.02841	1.98188	-1.23199
O	1.37943	0.84678	0.31306
C	0.05002	3.13490	-0.43060
C	-0.61992	4.30127	-0.78494
C	-1.38502	4.34753	-1.94974
C	-1.47158	3.21144	-2.75676
C	-0.80239	2.04416	-2.40629
H	-1.85420	-1.57780	-1.11096
H	0.26659	-3.30664	0.15267
H	-0.63867	-2.96937	-2.74903
H	-0.64327	-4.46201	-1.79985
H	1.92371	-1.56232	-0.22184
H	0.02104	-0.48209	-2.39363
H	3.09282	-2.44251	-2.10851
H	1.86453	-1.77543	-3.17701
H	1.42106	-4.14529	-3.47239
H	1.83572	-4.49146	-1.79008
H	0.64497	3.09064	0.47509
H	-0.54653	5.17707	-0.14788
H	-1.90808	5.25655	-2.22761
H	-2.06182	3.23573	-3.66721
H	-0.88405	1.18210	-3.06002
C	-2.89207	0.49935	-0.01299
C	-3.20437	1.95798	0.40124
H	-2.90339	2.66880	-0.37176
H	-4.25114	2.09956	0.66832
N	-1.78809	0.18643	0.89368
C	-1.52184	1.22470	1.75448
O	-0.62913	1.31577	2.57781
O	-2.40511	2.21034	1.56713
C	-4.05678	-0.45414	0.13980

C	-4.47761	-0.86343	1.40743
C	-4.74353	-0.89699	-0.98983
C	-5.57481	-1.70819	1.54040
H	-3.94025	-0.52684	2.29044
C	-5.84461	-1.74108	-0.85659
H	-4.41339	-0.58259	-1.97679
C	-6.26091	-2.14757	0.40828
H	-5.89439	-2.02551	2.52754
H	-6.37162	-2.08409	-1.74075
H	-7.11511	-2.80819	0.51356
H	-2.53472	0.48937	-1.04750

TS2 ($_{\text{Gb-Hba}}$) (charge = -1; spin = 2)

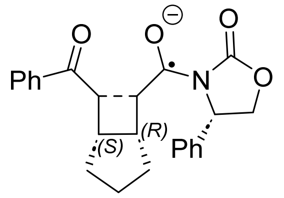

C	-0.88844	-2.07101	0.71086
C	-1.08094	-1.22221	1.82795
C	-0.03539	-1.31147	2.92387
C	-0.36993	-0.61781	4.24942
C	1.02152	-0.38485	2.29292
C	0.08368	0.41656	1.39288
C	0.35840	0.68474	0.03079
O	0.15114	-2.73493	0.49944
C	1.66719	0.43374	3.43487
C	0.99689	-0.10130	4.71953
Li	1.02659	-2.04300	-1.07534
Br	3.89496	1.99799	-0.07502
Br	3.45698	-2.35443	-1.30018
Li	2.86065	0.00488	-0.78692
C	-0.08859	1.93932	-0.62362
O	0.99810	-0.17085	-0.69911
C	-0.09800	2.00212	-2.02586
C	-0.47298	3.16857	-2.68439
C	-0.83043	4.30270	-1.95412
C	-0.80644	4.25795	-0.56015
C	-0.43646	3.09066	0.09917
H	-2.07779	-0.84665	2.03867
H	0.31156	-2.33576	3.09758
H	-1.04548	0.22885	4.07057
H	-0.85600	-1.28032	4.97044
H	1.74241	-0.93037	1.67911
H	-0.50117	1.16971	1.91644
H	2.75457	0.32563	3.46223
H	1.45309	1.50062	3.30197
H	0.92384	0.65044	5.50954
H	1.58017	-0.94024	5.11600
H	0.19328	1.11838	-2.58445
H	-0.48243	3.19594	-3.76955
H	-1.11598	5.21561	-2.46661
H	-1.06640	5.14014	0.01623
H	-0.39249	3.08818	1.18333
C	-3.33238	-1.85077	-0.06163
C	-3.90059	-2.54280	-1.31808
H	-4.74548	-2.01119	-1.75153
H	-4.15889	-3.58626	-1.12628
N	-1.90637	-2.09965	-0.28376
C	-1.65676	-2.37273	-1.60194
O	-0.57896	-2.44406	-2.16691

O	-2.81571	-2.51160	-2.26321
C	-3.68758	-0.38126	0.01434
C	-3.08096	0.54115	-0.83846
C	-4.67325	0.04804	0.90400
C	-3.45752	1.88044	-0.80494
H	-2.29529	0.22703	-1.52142
C	-5.05066	1.38862	0.93860
H	-5.14218	-0.66817	1.57380
C	-4.44369	2.30604	0.08318
H	-2.96469	2.59028	-1.46271
H	-5.81319	1.71614	1.63769
H	-4.73228	3.35173	0.11403
H	-3.66073	-2.36750	0.84336

TS2 ($_{Ga\text{-}Hab}$) (charge = -1; spin = 2)

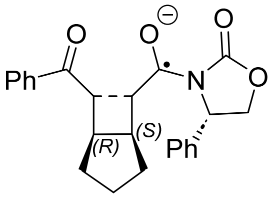

C	-0.87772	-0.08123	0.23197
C	-0.16317	-0.28549	1.45397
C	0.27959	-1.61673	2.06670
C	0.72917	-2.71438	1.07779
C	1.62974	-1.05070	2.56806
C	1.64937	0.09030	1.52384
C	2.16379	0.12773	0.17456
O	-1.06279	1.08289	-0.29083
C	2.60229	-2.23155	2.62024
C	2.07797	-3.26448	1.59650
Li	0.12938	1.12257	-1.93755
Br	0.51639	3.57659	2.16017
Br	0.20275	3.60146	-2.10139
Li	0.32935	2.39435	0.10936
C	3.13879	-0.84478	-0.39485
O	1.68930	1.00059	-0.60823
C	2.84423	-1.48886	-1.60279
C	3.75084	-2.39044	-2.14983
C	4.97312	-2.62307	-1.51750
C	5.28924	-1.94865	-0.33996
C	4.37192	-1.06452	0.22310
H	-0.45004	0.45981	2.19798
H	-0.38752	-2.01660	2.83909
H	0.88109	-2.27808	0.08720
H	-0.01752	-3.50489	0.96213
H	1.53689	-0.57212	3.54691
H	1.61916	1.09807	1.93870
H	2.54647	-2.65086	3.62984
H	3.64310	-1.95500	2.45222
H	2.77713	-3.39855	0.76504
H	1.94906	-4.24087	2.07095
H	1.89240	-1.29370	-2.09168
H	3.50896	-2.90872	-3.07202
H	5.68264	-3.32125	-1.94965
H	6.24942	-2.10886	0.13923
H	4.61752	-0.52351	1.13199
C	-2.33264	-2.15468	-0.07078
C	-2.31925	-3.13804	-1.25905
H	-1.63604	-3.97285	-1.08500
H	-3.30913	-3.50364	-1.52581
N	-1.30708	-1.21594	-0.52925

C	-1.12458	-1.31873	-1.86864
O	-0.43175	-0.60207	-2.58441
O	-1.80699	-2.36330	-2.35984
C	-3.68207	-1.50365	0.16462
C	-4.03568	-0.29490	-0.43662
C	-4.61385	-2.17405	0.96104
C	-5.31180	0.23254	-0.24332
H	-3.30730	0.25247	-1.02758
C	-5.88848	-1.64877	1.14860
H	-4.33758	-3.11155	1.43738
C	-6.24031	-0.44247	0.54452
H	-5.57743	1.17627	-0.70852
H	-6.60398	-2.17647	1.77072
H	-7.23202	-0.02819	0.69358
H	-2.00729	-2.65924	0.84280

TS2 ($_{Gb\text{-}Hbb}$) (charge = -1; spin = 2)

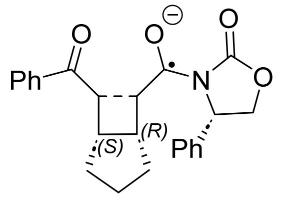

C	0.37780	-1.04710	0.42040
C	0.63280	-0.57230	-0.89770
C	1.72780	0.39710	-1.32790
C	2.05780	1.51120	-0.31430
C	0.84200	1.14290	-2.35370
C	-0.48220	0.76600	-1.65190
C	-1.19840	1.41620	-0.58650
O	-0.68380	-1.70150	0.74570
C	1.42050	2.55750	-2.46060
C	2.13060	2.83130	-1.11380
Li	-1.76450	-0.34740	1.86260
Br	-2.72240	-2.12260	-2.47830
Br	-3.90740	-1.58060	1.56690
Li	-2.26970	-1.26660	-0.31600
C	-1.03860	2.85110	-0.21370
O	-1.97870	0.71400	0.12330
C	-1.20040	3.85180	-1.17480
C	-1.09410	5.19320	-0.81500
C	-0.81820	5.53900	0.50610
C	-0.67610	4.54320	1.47370
C	-0.80490	3.20400	1.12080
H	0.36700	-1.31790	-1.64880
H	2.63680	-0.07240	-1.72450
H	1.25080	1.58210	0.42200
H	2.96990	1.30050	0.24790
H	0.83060	0.64660	-3.32840
H	-1.14850	0.11810	-2.22080
H	2.15780	2.54130	-3.26990
H	0.68630	3.32010	-2.72010
H	1.64430	3.64060	-0.55950
H	3.16790	3.13330	-1.28350
H	-1.43040	3.57460	-2.19930
H	-1.22860	5.96620	-1.56460
H	-0.72400	6.58360	0.78500
H	-0.46880	4.81330	2.50410
H	-0.69540	2.41940	1.86530
C	2.47760	-1.60150	1.75660
C	2.96410	-0.93920	3.04600
H	3.65420	-0.11320	2.86290

H	3.39540	-1.64060	3.75710
N	1.30000	-0.74060	1.48060
C	0.85220	-0.22920	2.65430
O	-0.22680	0.31780	2.86060
O	1.76480	-0.38370	3.62330
C	3.44030	-1.69910	0.59770
C	4.53930	-0.85440	0.43150
C	3.17230	-2.66180	-0.38100
C	5.34320	-0.95990	-0.70190
H	4.78250	-0.10220	1.17440
C	3.96860	-2.76390	-1.51710
H	2.32510	-3.33060	-0.25080
C	5.05640	-1.90810	-1.68090
H	6.19470	-0.29760	-0.81800
H	3.74270	-3.51160	-2.27000
H	5.68250	-1.98500	-2.56380
H	2.10390	-2.61000	1.98000

TS2 ($_{Ga\text{-}Hac}$) (charge = -1; spin = 2)

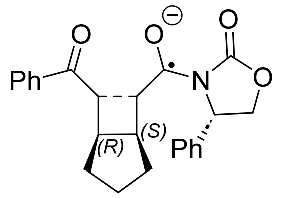

C	0.46200	0.00690	0.63780
C	-0.12950	-0.76510	-0.35900
C	0.09580	-0.64880	-1.86200
C	0.46530	-1.98510	-2.53680
C	-1.36360	-0.30140	-2.26610
C	-1.65280	0.47690	-0.99590
C	-2.63950	0.51300	0.02670
O	0.82810	1.21040	0.55660
C	-1.96790	-1.69250	-2.51270
C	-0.83570	-2.44140	-3.23590
Li	-0.74180	2.14950	1.45780
Br	3.30750	1.60080	-1.71030
Br	-0.21600	4.15440	0.13790
Li	1.50200	2.53990	-0.54700
C	-3.90720	-0.26650	-0.03710
O	-2.37660	1.14590	1.09260
C	-4.45830	-0.76930	1.14620
C	-5.65000	-1.48400	1.11890
C	-6.31740	-1.68780	-0.08860
C	-5.79030	-1.16400	-1.26710
C	-4.59390	-0.45440	-1.24020
H	-0.52300	-1.72360	-0.02050
H	0.81730	0.14930	-2.06380
H	0.79810	-2.70940	-1.78390
H	1.28920	-1.86290	-3.24440
H	-1.45030	0.31320	-3.17210
H	-1.00780	1.35340	-0.93400
H	-2.89380	-1.67370	-3.09350
H	-2.20150	-2.17740	-1.55740
H	-0.97400	-3.52670	-3.22880
H	-0.81710	-2.12550	-4.28590
H	-3.93110	-0.57930	2.07550
H	-6.06510	-1.88030	2.04100
H	-7.25110	-2.24200	-0.10880
H	-6.31810	-1.29890	-2.20650
H	-4.19170	-0.01800	-2.14970
C	1.19140	-1.87660	2.21970

C	1.77410	-1.57590	3.61340
H	1.70690	-2.41750	4.30300
H	2.81350	-1.24140	3.52590
N	0.59250	-0.57580	1.93870
C	0.44050	0.17030	3.07440
O	-0.11050	1.24280	3.19510
O	0.98090	-0.50030	4.12480
C	2.21590	-2.32410	1.20030
C	2.98340	-1.40430	0.47760
C	2.37440	-3.68850	0.95810
C	3.87660	-1.84880	-0.49200
H	2.87010	-0.33090	0.61390
C	3.29270	-4.13500	0.01040
H	1.76170	-4.40500	1.50120
C	4.03730	-3.21390	-0.72210
H	4.39830	-1.10100	-1.08180
H	3.40620	-5.19950	-0.17130
H	4.72970	-3.55940	-1.48370
H	0.40690	-2.64300	2.29530

TS2 ($_{Gb\text{-}Hbc}$) (charge = -1; spin = 2)

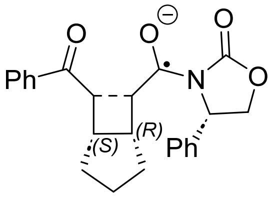

C	0.64965	-1.39649	-0.11438
C	0.12928	-0.75648	1.01679
C	0.84652	-0.15531	2.22414
C	0.33933	-0.66932	3.56689
C	0.24803	1.26500	2.05140
C	0.26239	1.18059	0.53573
C	-0.58665	1.55129	-0.54986
O	1.80061	-1.22002	-0.62740
C	-1.11821	1.17384	2.78321
C	-1.09379	-0.11090	3.65788
Li	1.25333	0.04073	-2.20071
Br	4.87159	-0.41613	1.52079
Br	3.28660	1.49391	-1.97087
Li	3.44372	-0.38327	-0.33693
C	-1.77438	2.43451	-0.43490
O	-0.30982	1.05647	-1.69264
C	-2.73640	2.41452	-1.45403
C	-3.83375	3.26763	-1.40913
C	-3.97771	4.16643	-0.35194
C	-3.00990	4.21614	0.65075
C	-1.91240	3.36231	0.60614
H	-0.92092	-0.95550	1.20242
H	1.93353	-0.18440	2.10281
H	0.37506	-1.75979	3.64887
H	0.96472	-0.25234	4.36382
H	0.86453	2.07835	2.45086
H	1.27979	1.04395	0.16982
H	-1.28494	2.06655	3.39087
H	-1.94196	1.12386	2.06520
H	-1.80870	-0.84186	3.26495
H	-1.38727	0.08994	4.69083
H	-2.60768	1.71591	-2.27485
H	-4.57718	3.23454	-2.19943
H	-4.83223	4.83443	-0.31710
H	-3.10382	4.93050	1.46229

H	-1.14434	3.42968	1.37049
C	-1.33063	-3.02283	-0.34448
C	-1.55093	-3.95128	-1.55703
H	-2.59748	-4.20491	-1.71542
H	-0.94198	-4.85538	-1.49151
N	-0.26897	-2.17543	-0.88591
C	-0.25091	-2.22930	-2.25214
O	0.38095	-1.53042	-3.02659
O	-1.09637	-3.17977	-2.68240
C	-2.59151	-2.28311	0.06636
C	-2.92495	-1.03167	-0.45492
C	-3.45063	-2.90264	0.97684
C	-4.11102	-0.41072	-0.06410
H	-2.24128	-0.52485	-1.13454
C	-4.63705	-2.28367	1.36048
H	-3.18543	-3.87197	1.39158
C	-4.96843	-1.03462	0.83958
H	-4.35732	0.56898	-0.46049
H	-5.29528	-2.77143	2.07198
H	-5.88777	-0.54469	1.14314
H	-0.94539	-3.58964	0.50707

TS2 ($_{Ga-Had}$) (charge = -1; spin = 2)

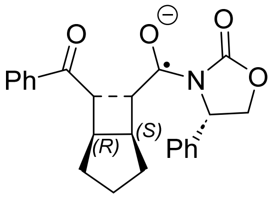

C	0.65168	0.38688	-0.12587
C	0.64769	0.95405	1.15152
C	1.23410	2.15780	1.85976
C	1.59422	3.42014	1.03540
C	-0.12490	2.52090	2.51584
C	-0.99025	2.08674	1.33205
C	-2.04582	1.13879	1.26428
O	-0.17080	-0.55177	-0.39365
C	-0.08473	4.02524	2.75965
C	0.69936	4.58183	1.55434
Li	-1.15403	-0.83273	-1.97252
Br	-0.05649	-2.73461	2.42008
Br	-2.61998	-2.54320	-0.92440
Li	-1.37707	-1.35026	0.96952
C	-2.94808	1.17099	0.06929
O	-2.17457	0.17922	2.08622
C	-4.21289	0.58063	0.18005
C	-5.08662	0.56024	-0.90104
C	-4.69738	1.09816	-2.12707
C	-3.42930	1.66464	-2.25875
C	-2.56590	1.71470	-1.16680
H	0.27031	0.22569	1.86903
H	2.03399	1.89907	2.56115
H	1.41229	3.26103	-0.03229
H	2.65493	3.65730	1.14828
H	-0.34691	1.91566	3.39896
H	-0.97843	2.81550	0.52465
H	0.45938	4.21727	3.69057
H	-1.07956	4.46973	2.85586
H	0.00884	4.90083	0.76837
H	1.28951	5.45951	1.82712
H	-4.48943	0.12691	1.12627

H	-6.06859	0.11038	-0.79273
H	-5.37341	1.07225	-2.97560
H	-3.11000	2.07166	-3.21326
H	-1.57645	2.14383	-1.29841
C	2.89472	1.08715	-1.05543
C	3.19485	1.62374	-2.46597
H	3.11282	2.71221	-2.50979
H	4.15529	1.29398	-2.85750
N	1.44926	0.89116	-1.18645
C	1.11173	0.73317	-2.50561
O	0.04416	0.37206	-2.97578
O	2.15238	1.06109	-3.28320
C	3.64497	-0.18985	-0.71589
C	3.04804	-1.45152	-0.74743
C	5.00035	-0.08033	-0.39157
C	3.80475	-2.58754	-0.45784
H	1.99029	-1.56225	-0.96860
C	5.75363	-1.21484	-0.11074
H	5.46679	0.90151	-0.35762
C	5.15524	-2.47418	-0.14327
H	3.32916	-3.56283	-0.47528
H	6.80476	-1.11639	0.13976
H	5.73911	-3.36053	0.08169
H	3.11396	1.85210	-0.30752

TS2 ($_{Gb\text{-}Hbd}$) (charge = -1; spin = 2)

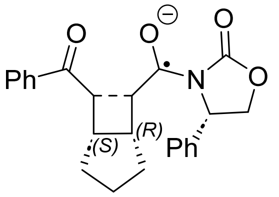

C	0.10880	0.44680	-0.41860
C	-0.07430	1.66400	-1.06300
C	0.48650	3.06440	-0.82610
C	1.31330	3.38610	0.43530
C	-0.88560	3.77710	-0.60280
C	-1.75090	2.55250	-0.35930
C	-2.77640	2.13520	-1.26810
O	-0.49430	-0.63660	-0.79750
C	-0.72390	4.63340	0.65830
C	0.30180	3.85090	1.48940
Li	-1.94580	-1.03060	0.47600
Br	2.04380	-3.34670	-1.63610
Br	-1.00940	-3.20780	1.29390
Li	0.04580	-2.42380	-0.79770
C	-3.67830	1.00630	-0.84110
O	-2.86160	2.56940	-2.43270
C	-4.21230	0.18020	-1.83820
C	-5.03450	-0.89240	-1.50830
C	-5.35660	-1.14360	-0.17360
C	-4.85420	-0.31130	0.82690
C	-4.01270	0.75250	0.49720
H	-0.45860	1.53910	-2.07380
H	1.02430	3.39200	-1.72070
H	1.94300	2.56380	0.76940
H	1.97510	4.22480	0.18980
H	-1.24650	4.32580	-1.47560
H	-1.80730	2.19460	0.66480
H	-0.31140	5.61440	0.39570
H	-1.67520	4.79380	1.17530
H	-0.17530	2.98520	1.96660

H	0.75910	4.44980	2.28130
H	-3.96290	0.39760	-2.87150
H	-5.42970	-1.53220	-2.29090
H	-6.00470	-1.97470	0.08440
H	-5.11770	-0.48570	1.86540
H	-3.63710	1.39600	1.28610
C	2.07240	-0.27600	1.08420
C	2.09170	-0.24670	2.61340
H	2.48750	0.69350	3.00510
H	2.60610	-1.09510	3.06030
N	0.75730	0.38070	0.87090
C	-0.03670	0.08680	1.93730
O	-1.26090	0.16100	1.97580
O	0.69860	-0.31400	2.98080
C	3.21040	0.39260	0.35040
C	4.19520	1.15410	0.98170
C	3.25330	0.24230	-1.04070
C	5.19090	1.77820	0.23150
H	4.20200	1.27490	2.05970
C	4.23950	0.87590	-1.78970
H	2.52190	-0.39790	-1.52790
C	5.20880	1.65080	-1.15440
H	5.95120	2.36630	0.73490
H	4.25750	0.75590	-2.86800
H	5.98110	2.14290	-1.73640
H	2.00170	-1.31570	0.72950

Intermediate Haa (charge = -1; spin = 2)

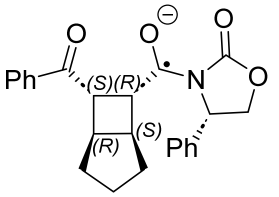

C	-0.90720	-0.54048	1.22060
C	-0.77358	-1.43638	0.03002
C	0.42389	-2.40768	0.02412
C	0.15024	-3.91732	-0.11899
C	0.81997	-1.86644	-1.37618
C	-0.18232	-0.68420	-1.25312
C	0.43398	0.63956	-0.91672
O	-0.24219	-0.62357	2.23857
C	0.43006	-2.99878	-2.33540
C	0.70730	-4.26338	-1.51234
Li	1.14065	0.98820	1.94196
Br	4.64493	-0.78239	-1.31608
Br	3.39901	0.36468	2.65106
Li	3.02054	-0.02274	0.22901
C	0.01061	1.89063	-1.46581
O	1.30113	0.62026	0.07342
C	0.59222	3.09216	-0.96420
C	0.18051	4.33396	-1.41637
C	-0.82167	4.45184	-2.38643
C	-1.38837	3.28535	-2.91215
C	-0.98343	2.03334	-2.47683
H	-1.73083	-1.91586	-0.19967
H	1.13358	-2.16399	0.81834
H	-0.92724	-4.11610	-0.08672
H	0.61329	-4.50718	0.67553
H	1.86356	-1.56591	-1.50410
H	-0.88862	-0.62077	-2.08324
H	0.97661	-2.96436	-3.28131

H	-0.64227	-2.94457	-2.56803
H	0.26470	-5.16660	-1.94081
H	1.79046	-4.42081	-1.44607
H	1.37368	3.00591	-0.21656
H	0.64345	5.22834	-1.00836
H	-1.14520	5.42722	-2.73302
H	-2.15372	3.35536	-3.68002
H	-1.43357	1.15515	-2.93026
C	-2.85920	0.60895	0.04608
C	-3.37865	2.03869	0.30184
H	-3.55817	2.58649	-0.62209
H	-4.27041	2.03796	0.93222
N	-1.79074	0.54489	1.05142
C	-1.44494	1.81749	1.47956
O	-0.47737	2.13402	2.14092
O	-2.32234	2.70328	1.01570
C	-3.91546	-0.45631	0.21478
C	-4.41931	-0.77002	1.47956
C	-4.40902	-1.11736	-0.90952
C	-5.41051	-1.73676	1.61412
H	-4.02840	-0.26245	2.35766
C	-5.40605	-2.08162	-0.77419
H	-4.00880	-0.87913	-1.89188
C	-5.90668	-2.39212	0.48726
H	-5.79572	-1.98008	2.59869
H	-5.78485	-2.59265	-1.65294
H	-6.67912	-3.14637	0.59464
H	-2.42683	0.54914	-0.95610

Intermediate Hba (charge = -1; spin = 2) *not completed converged

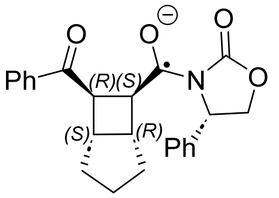

C	-0.19609	-0.71959	-1.71073
C	0.46113	0.62959	-1.68022
C	-0.43551	1.86451	-1.89366
C	-0.15365	2.77465	-3.10468
C	0.21450	2.52713	-0.64926
C	0.91032	1.18017	-0.27382
C	0.36929	0.48089	0.94476
O	-1.39561	-0.91218	-1.77944
C	1.10798	3.63204	-1.22797
C	0.33925	4.09067	-2.47469
Li	-1.09228	-2.15067	1.10853
Br	-3.91225	1.69527	-0.53379
Br	-3.51573	-2.01240	1.19768
Li	-2.35526	-0.05878	0.02844
C	0.88541	0.79977	2.23618
O	-0.63065	-0.35935	0.80965
C	0.34338	0.17595	3.39646
C	0.84957	0.44081	4.65817
C	1.90560	1.34279	4.83568
C	2.43391	1.98839	3.71128
C	1.93960	1.73593	2.44228
H	1.31952	0.62982	-2.35991
H	-1.49405	1.63337	-1.76678
H	0.63372	2.34378	-3.73451
H	-1.03384	2.91800	-3.73649

H	-0.47909	2.90728	0.10794
H	1.99895	1.27008	-0.19892
H	1.31115	4.43909	-0.51913
H	2.07456	3.20490	-1.53079
H	0.94187	4.69506	-3.15826
H	-0.52223	4.69507	-2.16707
H	-0.49096	-0.50736	3.27364
H	0.41357	-0.05506	5.52150
H	2.29788	1.54787	5.82597
H	3.24229	2.70476	3.83162
H	2.36851	2.26316	1.59546
C	2.10217	-1.80867	-2.10955
C	2.33282	-3.32879	-2.21371
H	3.32454	-3.63065	-1.88309
H	2.12893	-3.70455	-3.21780
N	0.69519	-1.81590	-1.66728
C	0.38330	-3.02728	-1.08830
O	-0.58274	-3.30118	-0.40368
O	1.36758	-3.90085	-1.31211
C	3.02941	-1.10495	-1.14331
C	2.95811	-1.35783	0.22932
C	3.95738	-0.18342	-1.62690
C	3.79936	-0.68244	1.10768
H	2.22941	-2.06196	0.62499
C	4.80183	0.49188	-0.74704
H	4.00922	0.01864	-2.69380
C	4.71824	0.24671	0.62169
H	3.71864	-0.86095	2.17483
H	5.51396	1.21512	-1.13034
H	5.36097	0.78392	1.31162
H	2.17564	-1.35519	-3.09990

*

Maximum force	Converged
RMS force	Converged
Maximum displacement	Not converged
RMS displacement	Not converged

Intermediate Hab (charge = -1; spin = 2)

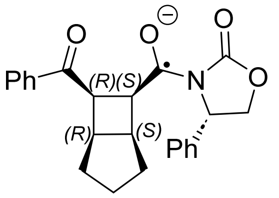

C	-0.93218	-0.09116	0.25536
C	-0.11777	-0.40028	1.45423
C	0.17913	-1.84615	1.89839
C	0.61339	-2.81919	0.77952
C	1.53680	-1.38752	2.47133
C	1.49019	-0.13867	1.53226
C	2.10479	-0.00939	0.17505
O	-1.02981	1.12118	-0.23494
C	2.48287	-2.58514	2.38488
C	1.93908	-3.47160	1.23968
Li	-0.02779	1.13528	-1.91871
Br	0.81312	3.45006	2.20060
Br	0.30883	3.59445	-2.03899
Li	0.36370	2.35190	0.15777
C	3.19515	-0.87653	-0.35179
O	1.66788	0.87409	-0.57101

C	4.38522	-1.04500	0.35702
C	5.41025	-1.81392	-0.18813
C	5.23661	-2.42814	-1.42687
C	4.05011	-2.25113	-2.13998
C	3.03618	-1.45790	-1.61442
H	-0.49956	0.17770	2.30686
H	-0.53112	-2.30518	2.59369
H	0.78997	-2.26425	-0.14870
H	-0.15693	-3.56111	0.55006
H	1.45873	-1.00871	3.49367
H	1.69125	0.81329	2.03642
H	2.40095	-3.12034	3.33660
H	3.53424	-2.32673	2.26227
H	2.64610	-3.53177	0.40632
H	1.77223	-4.49230	1.59387
H	4.51445	-0.55922	1.31985
H	6.34137	-1.93554	0.35490
H	6.03044	-3.04031	-1.84216
H	3.92017	-2.72601	-3.10665
H	2.10629	-1.30334	-2.15725
C	-2.56315	-2.03660	-0.08529
C	-2.62796	-2.99357	-1.29483
H	-2.02549	-3.89108	-1.13436
H	-3.64458	-3.26541	-1.57425
N	-1.47428	-1.16242	-0.52200
C	-1.27298	-1.27610	-1.85147
O	-0.50737	-0.61165	-2.55003
O	-2.04161	-2.24694	-2.37632
C	-3.86652	-1.30603	0.17724
C	-4.09309	-0.00374	-0.26940
C	-4.88646	-1.99201	0.84266
C	-5.33227	0.59980	-0.05428
H	-3.29288	0.54970	-0.75235
C	-6.12325	-1.39023	1.05076
H	-4.70932	-3.00407	1.19932
C	-6.34907	-0.08995	0.60023
H	-5.49970	1.61538	-0.39873
H	-6.90800	-1.93257	1.56818
H	-7.31135	0.38380	0.76492
H	-2.26664	-2.58680	0.81347

Intermediate Hbb (charge = -1; spin = 2)

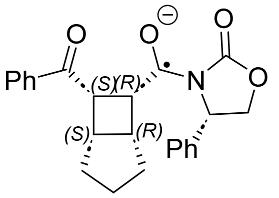

C	-1.13448	-0.80304	0.34417
C	-0.99119	0.67089	0.52854
C	-1.75560	1.66368	-0.37912
C	-1.97268	1.28446	-1.85133
C	-0.50105	2.57708	-0.42504
C	0.35366	1.40734	0.20364
C	1.34368	0.80108	-0.74454
O	-0.17916	-1.64905	0.65775
C	-0.29069	3.01646	-1.89073
C	-1.57973	2.56384	-2.60079
Li	0.58269	-2.01380	-1.07805
Br	2.57163	-0.03277	2.75704
Br	2.78749	-2.93890	-0.26348
Li	1.48963	-1.64524	1.42589

C	2.70581	1.41283	-0.79492
O	1.08445	-0.13673	-1.49465
C	2.92348	2.74324	-0.42240
C	4.20030	3.28903	-0.51273
C	5.26468	2.50254	-0.95090
C	5.05298	1.17018	-1.30633
C	3.77429	0.62842	-1.24235
H	-1.19727	0.94284	1.57671
H	-2.65992	2.09765	0.05800
H	-1.29340	0.46930	-2.13067
H	-2.99441	0.95284	-2.05594
H	-0.53309	3.42281	0.26510
H	0.90717	1.70261	1.09635
H	-0.11769	4.09225	-1.97557
H	0.57464	2.52052	-2.34410
H	-1.44355	2.41260	-3.67510
H	-2.36079	3.32190	-2.46770
H	2.09638	3.35664	-0.07609
H	4.36629	4.32514	-0.23760
H	6.26181	2.92698	-1.00976
H	5.88589	0.55595	-1.63244
H	3.59066	-0.41549	-1.47938
C	-3.58078	-1.53919	0.25108
C	-4.29645	-2.28331	-0.88078
H	-4.85775	-1.61756	-1.53891
H	-4.94512	-3.08189	-0.52535
N	-2.25782	-1.32331	-0.37557
C	-2.09026	-2.20615	-1.38610
O	-1.05841	-2.44104	-2.01182
O	-3.23261	-2.86807	-1.65224
C	-4.20897	-0.25487	0.74364
C	-5.01423	0.56341	-0.05195
C	-3.90153	0.15979	2.04255
C	-5.49679	1.77356	0.44230
H	-5.26900	0.27841	-1.06709
C	-4.36917	1.37488	2.53397
H	-3.27514	-0.47386	2.66594
C	-5.17052	2.18582	1.73226
H	-6.12416	2.39724	-0.18612
H	-4.11317	1.68483	3.54183
H	-5.54272	3.13152	2.11261
H	-3.44024	-2.20705	1.11111

Intermediate Hac (charge = -1; spin = 2)

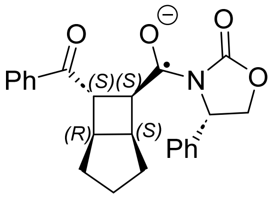

C	-1.24404	0.64040	-0.07865
C	-0.18648	1.70744	-0.18989

C	-0.12975	2.76960	-1.32240
C	-0.20968	4.21937	-0.84547
C	1.38949	2.44723	-1.51675
C	1.18947	1.19698	-0.60774
C	2.11134	0.82347	0.52034
O	-0.83670	-0.55925	0.27264
C	2.14641	3.65527	-0.93910
C	1.11859	4.43544	-0.09994
Li	-0.05098	-0.38528	2.04225
Br	1.49311	-2.30930	-2.17542
Br	1.03438	-2.65497	2.01465
Li	0.20396	-1.97519	-0.22251
C	3.53831	0.51441	0.23909
O	1.67124	0.72117	1.66437
C	4.39446	0.22670	1.30823
C	5.72650	-0.08481	1.06868
C	6.20779	-0.11627	-0.24117
C	5.35701	0.15911	-1.31031
C	4.02322	0.47602	-1.07293
H	-0.11639	2.21478	0.78586
H	-0.77519	2.52959	-2.17091
H	-1.08682	4.40219	-0.21528
H	-0.26565	4.89532	-1.70649
H	1.71989	2.20435	-2.52900
H	1.10108	0.30197	-1.23968
H	2.48388	4.28173	-1.77154
H	3.03748	3.37042	-0.36984
H	1.05959	4.01754	0.91113
H	1.37963	5.49168	0.00367
H	3.99587	0.25210	2.31703
H	6.39102	-0.30466	1.89744
H	7.24846	-0.36064	-0.42859
H	5.73242	0.12505	-2.32732
H	3.35630	0.67268	-1.90588
C	-3.75357	1.29103	0.06596
C	-4.49606	1.79190	1.32622
H	-4.49371	2.88278	1.38673
H	-5.51239	1.40938	1.40686
N	-2.42923	1.09188	0.63700
C	-2.48853	0.98910	1.98095
O	-1.57820	0.67757	2.74617
O	-3.72631	1.27871	2.42914
C	-4.36250	0.03458	-0.52943
C	-3.92723	-1.24188	-0.16809
C	-5.43055	0.16807	-1.42041
C	-4.55886	-2.36846	-0.69446
H	-3.06872	-1.35615	0.48706
C	-6.06197	-0.95722	-1.94152
H	-5.76706	1.16092	-1.70957
C	-5.62629	-2.23078	-1.57819
H	-4.21011	-3.35754	-0.41386
H	-6.88808	-0.84061	-2.63557
H	-6.11274	-3.11014	-1.98767
H	-3.72791	2.07908	-0.69251

Intermediate Hbc (charge = -1; spin = 2)

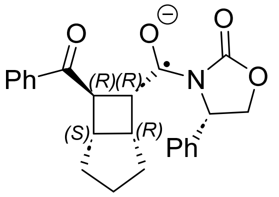

C	0.22638	1.40110	-1.15254
C	-0.50503	0.18685	-1.61032
C	-0.58240	-0.44188	-3.02786
C	-1.89481	-0.27692	-3.77530
C	-0.61860	-1.82470	-2.30525
C	0.16177	-1.14613	-1.15292
C	0.21014	-1.31515	0.33774
O	1.43273	1.56617	-1.23666
C	-2.13525	-2.09414	-2.11094
C	-2.92218	-0.99901	-2.88304
Li	1.31126	1.08491	2.05269
Br	3.90896	-1.06269	-1.68328
Br	3.77450	1.13565	1.80086
Li	2.52152	0.11448	-0.10028
C	-0.37695	-2.35651	1.11128
O	0.84779	-0.31071	0.90656
C	-0.45360	-2.23172	2.52748
C	-1.03272	-3.22373	3.30280
C	-1.54407	-4.38757	2.71805
C	-1.44930	-4.54217	1.32892
C	-0.87751	-3.55972	0.53939
H	-1.51432	0.20774	-1.20372
H	0.32800	-0.25907	-3.60421
H	-2.15793	0.76847	-3.96540
H	-1.81024	-0.77895	-4.74543
H	-0.10753	-2.66058	-2.78793
H	1.19693	-1.08807	-1.51708
H	-2.37872	-3.08889	-2.49274
H	-2.40828	-2.08828	-1.05137
H	-3.37287	-0.29211	-2.17719
H	-3.74320	-1.42025	-3.46795
H	-0.06196	-1.33083	2.98969
H	-1.08783	-3.09340	4.38033
H	-1.99372	-5.16208	3.32992
H	-1.81899	-5.45033	0.86092
H	-0.78633	-3.71896	-0.53031
C	-2.00230	2.59156	-0.65342
C	-2.04425	4.03412	-0.12083
H	-2.98617	4.29091	0.35901
H	-1.78448	4.76256	-0.89144
N	-0.56730	2.34206	-0.46744
C	-0.11438	3.09551	0.60169
O	0.88679	2.91350	1.26116
O	-1.01168	4.04364	0.88523
C	-2.91007	1.65671	0.13409
C	-2.42556	0.73404	1.06303
C	-4.28603	1.74008	-0.09853
C	-3.31107	-0.10165	1.74510
H	-1.35604	0.62739	1.23394
C	-5.16753	0.91225	0.58879
H	-4.66750	2.45048	-0.82832
C	-4.68015	-0.01326	1.51154
H	-2.91582	-0.83173	2.44540
H	-6.23307	0.98260	0.39705
H	-5.36649	-0.66727	2.03913
H	-2.25395	2.56591	-1.71647

Intermediate Had (charge = -1; spin = 2)

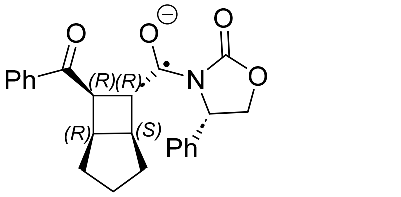

C	1.03685	0.39156	0.45565
C	-0.22727	1.07838	0.86439
C	-0.35955	2.44633	1.58202
C	0.64075	3.58466	1.31589
C	-1.59137	2.77771	0.68254
C	-1.20685	1.61522	-0.26166
C	-2.15197	0.56904	-0.77448
O	0.94473	-0.87929	0.12970
C	-1.35966	4.17152	0.09707
C	0.17330	4.28274	0.02943
Li	0.13587	-1.04972	-1.61318
Br	-1.95291	-2.13486	2.18909
Br	-0.23083	-3.55647	-1.38822
Li	-0.07387	-2.28976	0.75512
C	-3.62305	0.63191	-0.54939
O	-1.69584	-0.40307	-1.37548
C	-4.31135	-0.57431	-0.37479
C	-5.69025	-0.56638	-0.20238
C	-6.38937	0.64075	-0.23326
C	-5.71002	1.84114	-0.43311
C	-4.32571	1.84043	-0.57894
H	-0.78900	0.34152	1.44858
H	-0.54934	2.33774	2.65236
H	1.67843	3.25323	1.27152
H	0.56917	4.29361	2.14830
H	-2.55708	2.64145	1.17173
H	-0.62189	1.96643	-1.11951
H	-1.75572	4.93237	0.77910
H	-1.84091	4.30974	-0.87725
H	0.55832	3.75505	-0.85112
H	0.51877	5.31754	-0.03758
H	-3.74609	-1.50070	-0.37000
H	-6.22312	-1.49911	-0.05040
H	-7.46716	0.64496	-0.10603
H	-6.25717	2.77686	-0.47366
H	-3.79718	2.77413	-0.74476
C	3.31365	1.54920	0.04744
C	3.75089	2.28777	-1.22890
H	3.44098	3.33657	-1.21035
H	4.81581	2.20548	-1.43923
N	1.96580	1.15377	-0.37073
C	1.93967	1.03917	-1.72384
O	1.08389	0.49354	-2.41396
O	3.02432	1.61716	-2.27470
C	4.23245	0.39308	0.40254
C	4.03230	-0.90188	-0.07957
C	5.36010	0.66446	1.18270
C	4.94985	-1.90843	0.21974
H	3.13477	-1.13876	-0.64114
C	6.27800	-0.33945	1.47583
H	5.51851	1.67046	1.56423
C	6.07419	-1.63133	0.99324
H	4.77875	-2.91431	-0.15099
H	7.14718	-0.11534	2.08576
H	6.78449	-2.41816	1.22547
H	3.27347	2.24159	0.89032

Intermediate Hbd (charge = -1; spin = 2)

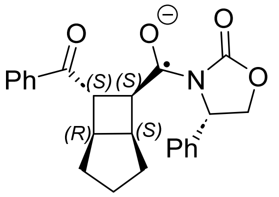

C	-1.05038	-0.69841	0.06012
C	-0.42230	0.62744	0.28792
C	-1.14634	1.97047	0.55914
C	-2.43241	2.36177	-0.20403
C	0.00133	2.70035	-0.20458
C	0.37851	1.35731	-0.87313
C	1.76927	0.83305	-1.06387
O	-0.31626	-1.78722	0.16984
C	-0.64476	3.74145	-1.11454
C	-1.99482	3.10477	-1.48092
Li	0.73221	-1.91456	-1.44806
Br	2.29496	-0.61846	2.67660
Br	2.49534	-3.49781	-0.39632
Li	1.20320	-2.10745	1.19053
C	2.98658	1.63969	-0.76556
O	1.91706	-0.32829	-1.44534
C	4.12917	0.96485	-0.31835
C	5.29642	1.67153	-0.05736
C	5.33774	3.05051	-0.26724
C	4.21115	3.72233	-0.73717
C	3.03252	3.02111	-0.97631
H	0.29548	0.48444	1.10313
H	-1.24839	2.18611	1.62576
H	-3.08016	1.51471	-0.42475
H	-3.00493	3.04370	0.43452
H	0.79222	3.08038	0.44561
H	-0.15901	1.20712	-1.81626
H	-0.80722	4.66804	-0.55207
H	-0.03941	3.98548	-1.99442
H	-1.86871	2.39742	-2.30809
H	-2.73199	3.84386	-1.80551
H	4.08063	-0.11006	-0.17698
H	6.17616	1.15003	0.30471
H	6.25168	3.60092	-0.06828
H	4.24914	4.79162	-0.91544
H	2.15927	3.54394	-1.35234
C	-3.46163	-1.33600	-0.49479
C	-4.08625	-1.46656	-1.88146
H	-4.51769	-0.52067	-2.22163
H	-4.81240	-2.27224	-1.97284
N	-2.13599	-0.79739	-0.89321
C	-1.85163	-1.29660	-2.12823
O	-0.75282	-1.36186	-2.66857
O	-2.96098	-1.76394	-2.72829
C	-4.20735	-0.49425	0.51522
C	-5.42811	0.11679	0.22353
C	-3.65599	-0.32563	1.79190
C	-6.07045	0.90857	1.17507
H	-5.89749	-0.01403	-0.74592
C	-4.28916	0.47613	2.73546
H	-2.71610	-0.81103	2.03721
C	-5.49765	1.10058	2.42805
H	-7.01853	1.37635	0.93074
H	-3.84236	0.60741	3.71573
H	-5.99348	1.72316	3.16562

H	-3.29834	-2.33653	-0.06772

Product Iaa (charge = 0; spin = 2)

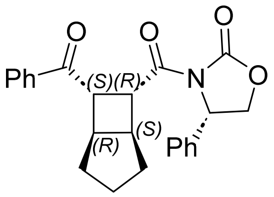

C	-0.50523	-0.55838	-0.27405
C	-1.09016	0.27151	0.82950
C	-1.15916	1.80330	0.54018
C	-2.51700	2.50605	0.70397
C	-0.33109	2.08372	1.82421
C	-0.10385	0.57340	2.02624
C	1.30610	0.06198	1.82371
O	0.58410	-0.27409	-0.77612
C	-1.30878	2.78258	2.78024
C	-2.25682	3.52415	1.82764
Li	1.94121	-1.51694	-1.64022
Br	1.92448	3.63598	-0.81147
Br	3.91159	-0.06103	-1.47917
Li	2.13378	1.29096	-0.48710
C	1.55056	-1.40552	1.90908
O	2.21672	0.82886	1.53251
C	2.83990	-1.87929	1.63643
C	3.08493	-3.24686	1.60417
C	2.04604	-4.14741	1.84410
C	0.76238	-3.68179	2.12716
C	0.51440	-2.31323	2.16442
H	-2.03410	-0.13112	1.19219
H	-0.64003	2.07121	-0.38332
H	-3.28755	1.78790	1.01064
H	-2.86133	2.97097	-0.22228
H	0.58629	2.65270	1.66168
H	-0.48652	0.17241	2.96966
H	-0.80761	3.44129	3.49309
H	-1.87694	2.04056	3.35560
H	-3.17319	3.86919	2.31301
H	-1.74477	4.40140	1.41511
H	3.62545	-1.16189	1.42432
H	4.08313	-3.61304	1.38915
H	2.23796	-5.21492	1.81439
H	-0.04238	-4.38216	2.32199
H	-0.48724	-1.96268	2.39825
C	-2.60272	-1.99996	-0.34786
C	-2.82848	-3.19878	-1.28803
H	-3.38379	-4.00722	-0.81883
H	-3.30733	-2.89103	-2.21962
N	-1.20516	-1.67069	-0.69636
C	-0.60561	-2.73976	-1.37022
O	0.56026	-2.85601	-1.67329
O	-1.50831	-3.67884	-1.60317
C	-3.58515	-0.87840	-0.59508
C	-3.44065	-0.02118	-1.68875
C	-4.67513	-0.73215	0.26313
C	-4.38335	0.97720	-1.91682
H	-2.59530	-0.13010	-2.36365
C	-5.62161	0.26252	0.02644
H	-4.78180	-1.39442	1.11787
C	-5.47491	1.11948	-1.06149

H	-4.26346	1.64542	-2.76290
H	-6.46707	0.37105	0.69726
H	-6.20701	1.89949	-1.24128
H	-2.64654	-2.32407	0.69710

Product Iba (charge = 0; spin = 2) *not completed converged

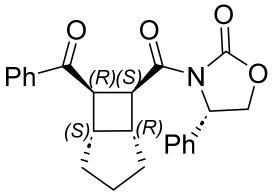

C	0.56671	-1.56890	-0.07298
C	0.18896	-0.98288	1.25095
C	1.30890	-0.19301	1.95854
C	1.21392	-0.12822	3.48844
C	0.69273	1.15528	1.49512
C	-0.59697	0.36601	1.07055
C	-1.11795	0.81322	-0.26931
O	1.53623	-1.19966	-0.71734
C	0.62062	2.08811	2.71533
C	1.46560	1.35733	3.77666
Li	0.72827	-0.25693	-2.39866
Br	4.77281	-0.49950	0.70459
Br	2.00394	1.68952	-2.18490
Li	3.11710	0.10133	-0.71263
C	-1.83983	2.11004	-0.33476
O	-0.86965	0.20431	-1.30567
C	-1.82618	2.79310	-1.55681
C	-2.46489	4.02046	-1.67194
C	-3.13109	4.56422	-0.57484
C	-3.15557	3.88404	0.64096
C	-2.50491	2.66207	0.76609
H	-0.33576	-1.71208	1.86921
H	2.33008	-0.41206	1.63463
H	0.20550	-0.40542	3.82952
H	1.92972	-0.79241	3.97762
H	1.17997	1.60443	0.62355
H	-1.38250	0.43030	1.82910
H	0.99487	3.08960	2.49498
H	-0.41501	2.19569	3.06091
H	1.21631	1.64922	4.79995
H	2.52625	1.57485	3.61128
H	-1.29375	2.35469	-2.39421
H	-2.43815	4.55717	-2.61398
H	-3.63058	5.52338	-0.66629
H	-3.67787	4.30816	1.49175
H	-2.53590	2.13678	1.71501
C	-1.36883	-3.24488	0.10169
C	-1.73479	-4.26896	-0.99132
H	-2.80347	-4.47301	-1.03676
H	-1.17032	-5.19830	-0.88385
N	-0.29356	-2.53227	-0.61113
C	-0.44973	-2.68455	-1.98936
O	0.06545	-2.03584	-2.86813
O	-1.34874	-3.64441	-2.22264
C	-2.51339	-2.34614	0.52742
C	-3.17484	-1.54405	-0.40606
C	-2.89568	-2.29403	1.86782
C	-4.17676	-0.67135	0.00704
H	-2.88841	-1.57008	-1.45429
C	-3.90552	-1.42799	2.28142

H	-2.38960	-2.92237	2.59752
C	-4.53859	-0.60704	1.35197
H	-4.66745	-0.03384	-0.72077
H	-4.19061	-1.39163	3.32755
H	-5.31559	0.07944	1.67164
H	-0.95317	-3.75726	0.97292

*

Maximum force	Converged
RMS force	Converged
Maximum displacement	Not converged
RMS displacement	Not converged

Product Iab (charge = 0; spin = 2)

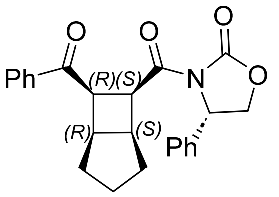

C	0.49359	-0.24044	0.79466
C	0.04144	-1.37087	-0.07974
C	-0.27207	-2.69273	0.73624
C	-0.18667	-2.72273	2.28007
C	-1.76721	-2.54273	0.36237
C	-1.40130	-1.44704	-0.66417
C	-2.35241	-0.28957	-0.85411
O	-0.22137	0.69004	1.16278
C	-2.43246	-2.07960	1.66541
C	-1.65267	-2.85476	2.73352
Li	0.25161	2.43368	2.10665
Br	1.48229	1.53155	-1.75947
Br	-1.06297	3.96204	0.63211
Li	-0.50258	1.94278	-0.59019
C	-3.81052	-0.59823	-0.90846
O	-1.98719	0.86747	-1.00332
C	-4.29559	-1.84206	-1.32737
C	-5.66681	-2.06862	-1.38053
C	-6.55515	-1.06527	-0.99665
C	-6.07506	0.17475	-0.57610
C	-4.70646	0.41254	-0.54299
H	0.80830	-1.49820	-0.84696
H	0.21932	-3.54799	0.26841
H	0.23731	-1.79991	2.69058
H	0.43963	-3.54418	2.63578
H	-2.24842	-3.43725	-0.03765
H	-1.30530	-1.89112	-1.66394
H	-3.50854	-2.26685	1.68120
H	-2.27895	-1.00021	1.80706
H	-1.81682	-2.47925	3.74591
H	-1.95452	-3.90753	2.71273
H	-3.61135	-2.62490	-1.63737
H	-6.04181	-3.02784	-1.72024
H	-7.62400	-1.24941	-1.02757
H	-6.76771	0.95394	-0.27703
H	-4.31476	1.37285	-0.22329
C	2.72812	-1.48378	0.96838
C	3.68649	-1.32387	2.16073
H	3.39215	-1.94174	3.01090
H	4.72604	-1.50167	1.89477
N	1.79091	-0.37217	1.26495

C	2.39355	0.54433	2.12510
O	1.98361	1.63216	2.46416
O	3.55474	0.05786	2.54831
C	3.40695	-1.36285	-0.37858
C	4.25430	-0.28645	-0.65151
C	3.21668	-2.35074	-1.34359
C	4.89564	-0.19693	-1.88050
H	4.41202	0.48977	0.09329
C	3.85366	-2.25766	-2.58005
H	2.57014	-3.19869	-1.12891
C	4.69274	-1.18032	-2.84851
H	5.54899	0.64409	-2.08696
H	3.69662	-3.02822	-3.32722
H	5.19095	-1.10625	-3.80949
H	2.20012	-2.43580	1.04232

Product Ibb (charge = 0; spin = 2)

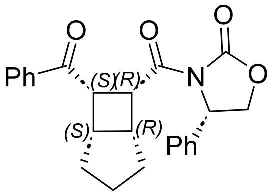

C	0.77995	0.22978	-0.39701
C	0.40631	-1.04661	0.29915
C	0.55201	-2.31705	-0.62107
C	0.58881	-2.07033	-2.13684
C	-0.91087	-2.74070	-0.29031
C	-1.06138	-1.47350	0.61211
C	-2.28636	-0.59848	0.51343
O	-0.00498	0.86274	-1.09956
C	-1.65176	-2.78537	-1.63272
C	-0.87358	-1.82617	-2.54034
Li	0.18949	2.52189	-2.22191
Br	0.59037	2.12308	2.16615
Br	-1.54485	3.87931	-1.05212
Li	-1.02839	2.05314	0.42435
C	-3.62219	-1.26290	0.54693
O	-2.24169	0.62379	0.48830
C	-3.80064	-2.56192	1.03635
C	-5.07346	-3.12079	1.07202
C	-6.16794	-2.39481	0.60446
C	-5.99385	-1.10190	0.11175
C	-4.72622	-0.53350	0.09133
H	1.00527	-1.14564	1.20292
H	1.34589	-2.98143	-0.27820
H	1.26477	-1.25903	-2.42745
H	0.95868	-2.98144	-2.61932
H	-1.01547	-3.66935	0.27246
H	-1.07777	-1.78674	1.66473
H	-1.57399	-3.80591	-2.02334
H	-2.71480	-2.54618	-1.55702
H	-1.16172	-0.79057	-2.33105
H	-1.05202	-2.01455	-3.60159
H	-2.95609	-3.13537	1.40381
H	-5.21120	-4.12291	1.46291
H	-7.15843	-2.83737	0.62491
H	-6.84578	-0.53874	-0.25355
H	-4.57187	0.47303	-0.28308
C	3.07971	0.05700	0.66217
C	4.25225	1.03729	0.49225

H	5.01779	0.64254	-0.17895
H	4.69515	1.32939	1.44201
N	2.11195	0.62387	-0.30195
C	2.49683	1.92591	-0.64704
O	1.89407	2.72902	-1.32357
O	3.68147	2.20211	-0.12040
C	3.46967	-1.37196	0.37448
C	3.82730	-1.76107	-0.91851
C	3.46997	-2.31083	1.40453
C	4.16097	-3.08591	-1.18134
H	3.82576	-1.03099	-1.72428
C	3.81101	-3.63657	1.14290
H	3.18918	-2.00741	2.40973
C	4.15008	-4.02609	-0.15096
H	4.42596	-3.38560	-2.18975
H	3.80388	-4.36386	1.94770
H	4.40614	-5.05998	-0.35703
H	2.64981	0.15989	1.66432

Product Iac (charge = 0; spin = 2)

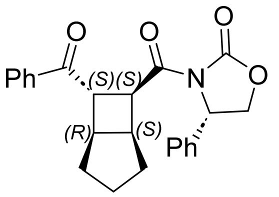

C	-1.14821	0.66403	-0.15666
C	-0.76182	-0.72251	-0.60473
C	-1.46374	-2.07568	-0.24754
C	-2.07595	-2.82232	-1.43110
C	-0.05435	-2.70152	-0.00772
C	0.53727	-1.26836	0.00564
C	1.81933	-0.83672	-0.66467
O	-0.35655	1.39106	0.41673
C	0.21013	-3.60935	-1.22456
C	-0.86045	-3.24786	-2.26897
Li	0.12356	3.42042	-1.84727
Br	2.69032	0.71994	2.21820
Br	1.95393	3.97442	-0.27262
Li	1.61640	1.62968	0.30154
C	3.04787	-1.66131	-0.57955
O	1.83498	0.25333	-1.23830
C	4.13381	-1.33315	-1.39846
C	5.31148	-2.06503	-1.31618
C	5.41390	-3.11588	-0.40395
C	4.33899	-3.43733	0.42391
C	3.15357	-2.71658	0.33295
H	-0.67675	-0.63152	-1.69717
H	-2.09007	-2.02383	0.64175
H	-2.79290	-2.22526	-2.00282
H	-2.60712	-3.70447	-1.05628
H	0.07400	-3.23536	0.93496
H	0.63236	-0.92164	1.04463
H	0.07371	-4.64828	-0.90909
H	1.22840	-3.52422	-1.61335
H	-0.51998	-2.41694	-2.89703
H	-1.08510	-4.07971	-2.94018
H	4.03592	-0.50523	-2.09253
H	6.15085	-1.81715	-1.95682
H	6.33584	-3.68419	-0.33613
H	4.42565	-4.24787	1.13912
H	2.32236	-2.95940	0.98682

C	-3.61886	0.43664	-0.82728
C	-4.59762	1.60783	-1.06122
H	-5.25777	1.44824	-1.91074
H	-5.17571	1.82356	-0.16059
N	-2.39023	1.19125	-0.53922
C	-2.51657	2.51475	-0.94703
O	-1.64946	3.35994	-1.01345
O	-3.76449	2.74421	-1.34054
C	-4.07902	-0.45096	0.30484
C	-3.80854	-0.12182	1.63465
C	-4.81534	-1.59827	0.01030
C	-4.24153	-0.95911	2.65931
H	-3.24724	0.77774	1.87229
C	-5.25439	-2.42984	1.03736
H	-5.03164	-1.85124	-1.02415
C	-4.95937	-2.11656	2.36241
H	-4.01558	-0.70747	3.69002
H	-5.81775	-3.32619	0.80083
H	-5.28981	-2.77069	3.16223
H	-3.48819	-0.14717	-1.74506

Product Ibc (charge = 0; spin = 2)

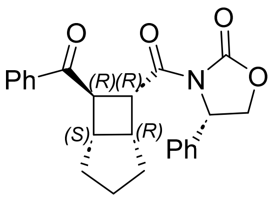

C	-1.02322	0.55832	-0.84887
C	-0.62542	-0.87471	-0.62213
C	-0.95361	-2.06095	-1.58816
C	-1.84508	-3.16934	-1.02913
C	0.51886	-2.55257	-1.41154
C	0.87011	-1.16979	-0.80518
C	1.74364	-0.91045	0.40037
O	-0.19976	1.46290	-0.85152
C	0.43411	-3.77209	-0.47031
C	-0.99866	-3.78668	0.09206
Li	-1.11390	3.01504	1.23796
Br	3.42749	1.44515	-1.33345
Br	1.17811	3.88345	1.21432
Li	1.52713	1.75619	0.06525
C	3.01656	-1.64058	0.61004
O	1.39642	-0.02049	1.17729
C	3.69467	-1.46545	1.82183
C	4.90883	-2.10388	2.03744
C	5.45845	-2.90752	1.03762
C	4.79423	-3.07309	-0.17680
C	3.57198	-2.44545	-0.39053
H	-0.95587	-1.11149	0.39840
H	-1.20164	-1.73076	-2.59931
H	-2.82199	-2.82700	-0.67931
H	-2.01193	-3.90918	-1.81982
H	1.06028	-2.78134	-2.33053
H	1.28296	-0.52183	-1.59155
H	0.59878	-4.67499	-1.06594
H	1.19634	-3.76231	0.31312
H	-1.06780	-3.17834	1.00123
H	-1.32899	-4.79269	0.35978
H	3.25582	-0.82572	2.57987
H	5.43092	-1.97408	2.97926
H	6.40928	-3.40311	1.20459

H	5.22880	-3.68969	-0.95589
H	3.06421	-2.56549	-1.34170
C	-3.49234	-0.06975	-1.14239
C	-4.50072	0.89297	-1.79105
H	-5.53265	0.68535	-1.51738
H	-4.38297	0.94222	-2.87465
N	-2.36913	0.86091	-0.98268
C	-2.87452	2.16065	-0.84553
O	-2.30600	3.12886	-0.39100
O	-4.14215	2.17614	-1.23716
C	-3.98611	-0.63721	0.17841
C	-3.46180	-0.24677	1.40905
C	-5.02213	-1.57663	0.14146
C	-3.96351	-0.79257	2.59141
H	-2.65844	0.48258	1.46251
C	-5.52168	-2.11891	1.31932
H	-5.43207	-1.89100	-0.81567
C	-4.99159	-1.72744	2.54929
H	-3.54532	-0.48227	3.54301
H	-6.32240	-2.84951	1.27819
H	-5.37941	-2.15237	3.46882
H	-3.23190	-0.86809	-1.83939

Product Iad (charge = 0; spin = 2)

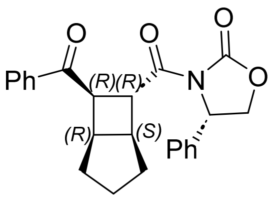

C	0.88653	0.17731	1.02082
C	0.11683	1.04102	0.06629
C	0.17143	2.59192	0.24849
C	0.59427	3.13110	1.62409
C	-1.39150	2.63616	0.23452
C	-1.40411	1.07584	0.30651
C	-2.19096	0.25723	-0.69101
O	0.51167	0.00570	2.17285
C	-1.80214	3.37804	1.50926
C	-0.66722	3.07907	2.49789
Li	0.48290	-1.78334	3.01313
Br	1.28338	-3.10617	-1.54016
Br	-1.42969	-2.60059	1.57017
Li	-0.79515	-2.27217	-0.77261
C	-3.64920	0.46467	-0.86415
O	-1.61392	-0.60364	-1.35254
C	-4.30565	-0.22913	-1.88945
C	-5.67135	-0.06584	-2.07498
C	-6.39135	0.78379	-1.23310
C	-5.74583	1.46801	-0.20532
C	-4.37594	1.31158	-0.02213
H	0.33567	0.76094	-0.96554
H	0.69893	3.08466	-0.57001
H	1.43859	2.58641	2.05698
H	0.91465	4.17047	1.49569
H	-1.84721	3.02953	-0.67683
H	-1.66786	0.71082	1.30870
H	-1.83571	4.45181	1.29360
H	-2.78382	3.09205	1.89702
H	-0.78776	2.07669	2.92193
H	-0.63429	3.78931	3.32731
H	-3.72873	-0.88557	-2.53175

H	-6.17805	-0.59822	-2.87253
H	-7.45923	0.91027	-1.37841
H	-6.30771	2.12226	0.45202
H	-3.87737	1.83828	0.78227
C	2.70309	-0.12854	-0.73194
C	3.95278	-1.03106	-0.61523
H	4.85886	-0.45603	-0.41769
H	4.07430	-1.66437	-1.49241
N	2.03664	-0.43429	0.54427
C	2.61468	-1.54099	1.15883
O	2.20952	-2.13865	2.13390
O	3.71759	-1.88796	0.51458
C	3.01716	1.33793	-0.90402
C	3.55713	2.08527	0.14520
C	2.75736	1.95375	-2.12778
C	3.81640	3.44205	-0.02498
H	3.76156	1.61017	1.10208
C	3.02780	3.30967	-2.30201
H	2.33115	1.37256	-2.94124
C	3.55056	4.05638	-1.24832
H	4.22349	4.02004	0.79810
H	2.82021	3.78294	-3.25584
H	3.75100	5.11456	-1.37902
H	2.07698	-0.49141	-1.55129

Product Ibd (charge = 0; spin = 2)

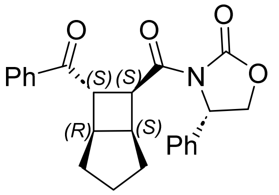

C	0.88653	0.17731	1.02082
C	0.11683	1.04102	0.06629
C	0.17143	2.59192	0.24849
C	0.59427	3.13110	1.62409
C	-1.39150	2.63616	0.23452
C	-1.40411	1.07584	0.30651
C	-2.19096	0.25723	-0.69101
O	0.51167	0.00570	2.17285
C	-1.80214	3.37804	1.50926
C	-0.66722	3.07907	2.49789
Li	0.48290	-1.78334	3.01313
Br	1.28338	-3.10617	-1.54016
Br	-1.42969	-2.60059	1.57017
Li	-0.79515	-2.27217	-0.77261
C	-3.64920	0.46467	-0.86415
O	-1.61392	-0.60364	-1.35254
C	-4.30565	-0.22913	-1.88945
C	-5.67135	-0.06584	-2.07498
C	-6.39135	0.78379	-1.23310
C	-5.74583	1.46801	-0.20532
C	-4.37594	1.31158	-0.02213
H	0.33567	0.76094	-0.96554
H	0.69893	3.08466	-0.57001
H	1.43859	2.58641	2.05698
H	0.91465	4.17047	1.49569
H	-1.84721	3.02953	-0.67683
H	-1.66786	0.71082	1.30870
H	-1.83571	4.45181	1.29360
H	-2.78382	3.09205	1.89702
H	-0.78776	2.07669	2.92193

H	-0.63429	3.78931	3.32731
H	-3.72873	-0.88557	-2.53175
H	-6.17805	-0.59822	-2.87253
H	-7.45923	0.91027	-1.37841
H	-6.30771	2.12226	0.45202
H	-3.87737	1.83828	0.78227
C	2.70309	-0.12854	-0.73194
C	3.95278	-1.03106	-0.61523
H	4.85886	-0.45603	-0.41769
H	4.07430	-1.66437	-1.49241
N	2.03664	-0.43429	0.54427
C	2.61468	-1.54099	1.15883
O	2.20952	-2.13865	2.13390
O	3.71759	-1.88796	0.51458
C	3.01716	1.33793	-0.90402
C	3.55713	2.08527	0.14520
C	2.75736	1.95375	-2.12778
C	3.81640	3.44205	-0.02498
H	3.76156	1.61017	1.10208
C	3.02780	3.30967	-2.30201
H	2.33115	1.37256	-2.94124
C	3.55056	4.05638	-1.24832
H	4.22349	4.02004	0.79810
H	2.82021	3.78294	-3.25584
H	3.75100	5.11456	-1.37902
H	2.07698	-0.49141	-1.55129

***i*Pr$_2$NEt (charge = 0; spin = 1)**

N	-0.00401	0.27285	-0.22387
C	0.29881	1.46160	0.56686
C	1.04005	-0.76390	-0.19467
C	-1.38214	-0.20862	-0.05898
C	-0.30094	2.72998	-0.03418
C	1.67189	-1.03808	1.17965
C	2.12706	-0.42572	-1.21643
C	-1.63457	-1.06787	1.18753
C	-1.85817	-0.93070	-1.32016
H	-0.02063	1.35750	1.61940
H	1.38580	1.58414	0.59104
H	0.56055	-1.69274	-0.52359
H	-1.99760	0.69269	0.04091
H	-0.01426	3.60437	0.55678
H	0.06194	2.86387	-1.05665
H	-1.39322	2.69704	-0.06446
H	2.32593	-1.91279	1.11619
H	2.28588	-0.19473	1.51072
H	0.91924	-1.23181	1.94660
H	2.89713	-1.20278	-1.24170
H	1.69263	-0.32541	-2.21392
H	2.61716	0.52055	-0.96164
H	-2.70327	-1.27951	1.28736
H	-1.11071	-2.02719	1.11841
H	-1.30605	-0.55978	2.09922
H	-2.92165	-1.17456	-1.24183
H	-1.70500	-0.29884	-2.19839
H	-1.31772	-1.87014	-1.47515

***i*Pr$_2$NEt radical cation (charge = -1; spin = 2)**

N	-0.00061	0.10869	0.25757
C	0.57315	1.19340	1.03210
C	0.85880	-0.89132	-0.37659
C	-1.45730	0.03379	0.12627
C	0.39141	2.53839	0.31215

C	1.55220	-1.73155	0.70583
C	1.85714	-0.21349	-1.32066
C	-1.96579	-1.28755	0.71451
C	-1.86385	0.21956	-1.34080
H	0.05018	1.20877	1.99471
H	1.62801	0.98452	1.20494
H	0.19967	-1.53876	-0.95704
H	-1.85265	0.86211	0.71885
H	0.83043	3.31415	0.94083
H	0.90167	2.53590	-0.65248
H	-0.66377	2.77360	0.16213
H	2.10951	-2.52350	0.20179
H	2.25605	-1.13471	1.28944
H	0.82450	-2.18959	1.37795
H	2.44396	-0.99843	-1.80139
H	1.34369	0.35833	-2.09619
H	2.54570	0.44148	-0.78124
H	-3.05649	-1.26923	0.67426
H	-1.61869	-2.14746	0.13715
H	-1.65829	-1.39871	1.75595
H	-2.95469	0.23361	-1.38295
H	-1.48871	1.16361	-1.74102
H	-1.51033	-0.60380	-1.96553

S7. Bibliography

[1] Romano, C.; Fiorito, D.; Mazet, C., J. Am. Chem. Soc. 2019, 141 (42), 16983–16990

[2] Richerds, E. L.; Murphy, P. J., Dinon, F., Fratucello, S.; Brown, P. M.; Gelbrich, T.; Hursthouse, M. B. Tetrahedron 2001, 57 (36), 7771-7784. [3] Shi, M., Zhang X.-N., *Eur. J. Org. Chem.* **2012**, 6271-6279.

[3] Nehate, Sagar P.; Godbole, Himanshu M.; Singh, Girij P.; Mathew, Jessy E.; Shenoy, Gautham G.[Synthetic Communications, 2019, vol. 49, # 9, p. 1173 – 1180.

[4] De Schutter, C.; Sari, O.; Coats, S. J.; Amblard, F.; Schinazi, R. F., J. Org. Chem. 2017, 82 (24), 13171–13178.

[5] 6 Suzuki, M.; Yamazaki, T.; Ohta, H.; Shima, K.; Ohi, K.; Nishiyama, S.; Sugai, T., Synlett 2000, 2000 (2), 189–192.

[6] Medici, F.; Resta, S.; Presenti, P.; Caruso, L.; Puglisi, A.; Raimondi, L.; Rossi, S.; Benaglia, M., *Eur. J. Org. Chem.* **2021**, *32*, 4521-452.

[7] Y. Roh, H. Y. Jang, V. Lynch, N. L. Bauld, M. J. Krische, *Org. Lett.* **2002**, *4,* 611-613.

[8] G. A. N. Felton, N. L. Bauld, Tetrahedron 2004, 60, 10999-11010.

[9] T. A. Halgren, J. Comput. *Chem.* **1999**, *20*, 720-729.

[10] Schrödinger Release 2024-1: MacroModel, Schrödinger, LLC, New York, NY, **2024**.

[11] Y. Zhao, D. G. Truhlar, *Theor. Chem. Acc.* **2007**, *120*, 215-241.

[12] Gaussian 16, Revision C.01, M. J. Frisch, G. W. Trucks, H. B. Schlegel, G. E. Scuseria, M. A. Robb, J. R. Cheeseman, G. Scalmani, V. Barone, G. A. Petersson, H. Nakatsuji, X. Li, M. Caricato, A. V. Marenich, J. Bloino,

B. G. Janesko, R. Gomperts, B. Mennucci, H. P. Hratchian, J. V. Ortiz, A. F. Izmaylov, J. L. Sonnenberg, D. Williams-Young, F. Ding, F. Lipparini, F. Egidi, J. Goings, B. Peng, A. Petrone, T. Henderson, D. Ranasinghe, V. G. Zakrzewski, J. Gao, N. Rega, G. Zheng, W. Liang, M. Hada, M. Ehara, K. Toyota, R. Fukuda, J. Hasegawa, M. Ishida, T. Nakajima, Y. Honda, O. Kitao, H. Nakai, T. Vreven, K. Throssell, J. A. Montgomery, Jr., J. E. Peralta, F. Ogliaro, M. J. Bearpark, J. J. Heyd, E. N. Brothers, K. N. Kudin, V. N. Staroverov, T. A. Keith, R. Kobayashi, J. Normand, K. Raghavachari, A. P. Rendell, J. C. Burant, S. S. Iyengar, J. Tomasi, M. Cossi, J. M. Millam, M. Klene, C. Adamo, R. Cammi, J. W. Ochterski, R. L. Martin, K. Morokuma, O. Farkas, J. B. Foresman, and D. J. Fox, Gaussian, Inc., Wallingford CT, **2019**.

[13] J. Tomasi, B. Mennucci, R. Cammi, *Chem Rev* **2005**, *105*, 2999-3093.

[14] Sheldrick, G. M., Crystal structure refinement with SHELXL. *Acta Crystallogr. C Struct. Chem.* **2015,** 71, (Pt 1), 3-8.
